# A squamous epithelial gene interaction perturbation network index for risk stratification in esophageal squamous cell carcinoma

**DOI:** 10.7150/jca.132865

**Published:** 2026-04-08

**Authors:** Chen Zhang, Huimin Wang, Huaping Wang, Xuexin Wang, Shan Tang, Feng Li, Li-dong Wang, Jianqiu Sheng

**Affiliations:** 1 Medical School of Chinese PLA, Beijing, 100853, China.; 2 Senior Department of Gastroenterology, the First Medical Center, Chinese PLA General Hospital, Beijing, 100853, China.; 3 Department of Gastroenterology, the Seventh Medical Center, Chinese PLA General Hospital, Beijing, 100700, China.; 4 Department of Oncology, the First Affiliated Hospital of Zhengzhou University, Zhengzhou, Henan, 450052, China.; 5 Department of Thoracic Surgery, the First Affiliated Hospital of Zhengzhou University, Zhengzhou, Henan, 450052, China.; 6 Henan Key Laboratory for Esophageal Cancer Research and State Key Laboratory of Metabolic Dysregulation & Prevention and Treatment of Esophageal Cancer of the First Affiliated Hospital of Zhengzhou University, Zhengzhou, Henan, 450052, China.

**Keywords:** esophageal squamous cell carcinoma, gene interaction perturbations, single-cell RNA sequencing, risk stratification, machine learning

## Abstract

Esophageal squamous cell carcinoma (ESCC) exhibits substantial molecular heterogeneity and unfavorable clinical outcomes. Current transcriptomic advances are shifting the focus from static gene expression profiles to the dynamic architecture of gene interaction networks. However, gene interaction perturbation signatures specific to ESCC remain poorly understood. This study aimed to develop a network-informed prognostic index derived from malignant epithelial cell signatures. In-house single-cell RNA sequencing data from 15 ESCC samples from the First Affiliated Hospital of Zhengzhou University were analyzed to identify dysregulated genes in malignant squamous epithelial cells. Then, a gene interaction perturbation network index (GIPNI) was constructed by systematically evaluating 75 combinations of machine-learning methods and validated across 3 independent cohorts. Associations between the GIPNI and genomic alterations, immune-related characteristics, and therapeutic response were also evaluated. Results showed ESCCs with high-GIPNI scores were associated with advanced clinicopathological features and overactivated mitotic cell cycle and epithelial cell differentiation pathways. Immune profiling suggested that low-GIPNI tumors had a more immune-infiltrated microenvironment. Notably, high-GIPNI ESCCs were associated with higher sensitivity to some common chemotherapeutic agents. Overall, the GIPNI provides a network-informed and malignant squamous cell-oriented framework for prognostic assessment in ESCC. This integrative approach may facilitate risk stratification and provide insights into individualized therapeutic strategies.

## Introduction

Esophageal squamous cell carcinoma (ESCC) is a major subtype of esophageal cancer and exhibits particularly high incidence and mortality rates in East Asia 1, 2. In terms of pathogenesis, ESCCs have driver gene mutations such as TP53, TTN, and NOTCH1, along with chromosomal instability and an immunosuppressive tumor microenvironment 3, 4. Although treatment options for advanced ESCC include targeted therapy, CAR-T cell therapy, and immunotherapy, only a subset of patients benefits from these treatments due to the molecular heterogeneity 5-8. Recent studies have advanced the understanding of ESCC progression and the identification of therapeutic targets based on multi-omics analyses 9-11.

Currently, transcriptomic advances are shifting the focus from static gene expression profiles to the dynamic architecture of gene interaction networks 12-15. Traditional analytical methods usually regarded gene expression as a steady-state snapshot 13. However, studies have shown that in normal tissues, a balanced feedback loop maintains a stable state, but this balance is broken and replaced by abnormal and over interconnected pathways 15, 16. Analyzing the dynamic networks with the hub nodes can provide a valuable perspective for understanding the heterogeneity and progression of ESCC. However, gene interaction perturbation signatures specific to ESCC remain poorly understood. In addition, conducting the construction of gene crosstalk networks within specific cell types is crucial to enhance their biological interpretability. Compared to bulk RNA sequencing, single-cell RNA sequencing (scRNA-seq) can delineate cell type-specific landscapes in ESCC 17-19. By analyzing gene expression patterns in specific cell types, it can distinguish the signals from malignant squamous epithelial cells from the background noise of other components in the tumor microenvironment 20. This high-resolution analysis enables the identification of signaling pathways that are intrinsically driven by cancer cells, providing a more solid foundation for discovering potential therapeutic targets 21. These technologies provide the basis for investigations of epithelial gene interaction perturbation signatures in ESCC.

In this study, we generated scRNA-seq data from 15 ESCC in-house samples, including tumor, para-cancerous, and normal tissues to characterize the dysregulated genes specific to the malignant squamous epithelial cells. Then, we constructed the gene interaction perturbation network and employed multiple machine learning algorithms to establish the gene interaction perturbation network index (GIPNI). This index was validated in multi-independent cohorts for its association with prognosis and other clinical features. We also identified multi-omics features, enriched biological pathways, and differential treatment sensitivities based on GIPNI. Notably, RAP1B, a key gene in the model, may serve as a robust prognostic biomarker in ESCC.

## Martials and Methods

### Data collection and preprocessing

In-house single cell sequencing data were generated from a total of 15 ESCC bio-samples, including 6 tumor, 6 paratumoral, and 3 normal tissues (distant paratumoral tissues). The transcriptomic data in this research comprised multiple sources. Normal esophageal squamous epithelial data were downloaded from the GTEx database (www.gtexportal.org) 22. Esophageal squamous cell carcinoma (ESCC) datasets were obtained from TCGA-ESCC within the TCGA-ESCA cohort (recognized by clinical information), as well as GSE53624, GSE53622, GSE104958, and GSE130078. For cross cancer validation of model genes, head and neck squamous cell carcinoma (HNSC) data were obtained from TCGA-HNSC, E-MTAB-8588, GSE75638, GSE117973, and GSE65858. Genomic data for the TCGA-ESCC cohort was acquired through the TCGAbiolinks package from the cancer genome atlas (TCGA) database.

RNA expression profiles derived from next-generation sequencing were transformed into transcripts per million (TPM) and subjected to log2 transformation. Prior to the development of the prognostic model, all gene expression values were standardized using Z-score scaling through the scale() function in R software to ensure that most expression levels remained within a range of -2 to 2. For model construction and validation, each cohort was processed independently. Specifically, the GSE53624 dataset was regarded as the training set, while all other cohorts were utilized as independent validation sets.

### Workflow of single-cell RNA sequencing

Single-cell RNA sequencing was applied using a microfluidic-based workflow. Fresh tissue samples were collected from the First Affiliated Hospital of Zhengzhou University and dissociated immediately into single-cell suspensions. After dissociation, the cell suspension was filtered with a cell sieve, and the quality was evaluated to ensure that the cells were qualified. The cell concentration was subsequently adjusted to make it suitable for efficient single-cell capture. The cell suspension was added to the microfluidic chip, and individual cells were separated into independent micropores. At this time, magnetic beads with cell specific barcode and unique molecular identifier were added. After lysis, the magnetic beads were recovered, reverse transcription reaction was performed, and cDNA was amplified. The amplified products were fragmented and ligated to construct the sequencing library. Finally, the library preparation was completed according to the standard process and sequenced on the Illumina platform.

### Single-cell RNA and squamous epithelial subcluster analysis

In-house single-cell sequencing data was analyzed by Seurat R package. The followed criteria were used to filter the low-quality cells: min features = 200, max features = 6000, min counts = 500, max counts = 40000, and max percent mt = 20. After filtering these cells, gene expression matrixes were normalized and scaled by default parameters. Principal component analysis (PCA) was performed to evaluate the major sources of variation. Harmony algorithm was used to correct the batch effects. Cell clustering was conducted with the Louvain method based on the first 50 principal components. Low-dimensional representations were generated using the Uniform Manifold Approximation and Projection (UMAP) to visualize the overall cellular landscape. Cell identity was assigned through manual inspection of canonical marker gene expression. These marker gene were typically. Squamous epithelial cells were subsequently isolated from the full dataset and subjected to an additional round of batch effects removing and clustering to resolve heterogeneity within this population. Copy number variation (CNV) scores for squamous epithelial cells were estimated using fastCNV, with T cells serving as the reference population 23. The malignment squamous epithelial cells was determined by integrating multiple lines of evidence, including epithelial marker expression, cluster-level transcriptional characteristics, and CNV profiles inferred from single-cell transcriptomic data.

### Construction of the squamous epithelial gene interaction perturbation network

The gene interaction perturbation network was constructed based on a previously described strategy 13, with minor adaptations. The high-confidence STRING interaction network (confidence score: 0.8) was used as a biologically informed prior knowledge framework for defining candidate gene-gene relationships as the previous study 24. The detailed result was displayed in Supplementary Table 2. Gene expression values were converted into relative ranks within each sample. We used rank-based differences rather than absolute expression values because rank information is generally more robust to platform-dependent scaling effects, technical noise, and batch variation across datasets. In addition, the relative ordering of two interacting genes can capture changes in gene-gene coordination and may therefore better reflect perturbation of biological interactions in a sample-specific manner 25. For gene pairs which were included in the background gene interaction perturbation network, rank differences between the two genes were calculated separately for each sample. These differences were used as a simple representation of the relative expression relationship between interacting genes. Because gene interactions tend to remain stable under normal physiological conditions, a reference pattern was generated using rank differences derived from genes ordered by their average expression levels in normal samples. In this step, normal esophageal squamous epithelial samples from the GTEx database were regarded as the reference dataset. For each sample, deviations from the reference rank-difference pattern were calculated and treated as indicators of interaction perturbation. These values were assembled into an edge-level perturbation matrix, which captured sample-specific changes in gene interactions while keeping the overall network structure unchanged. This matrix was subsequently used to assess the extent of interaction dysregulation across esophageal squamous cell carcinoma samples in comparison with the normal reference.

### Development of gene interaction perturbation network index

The gene interaction perturbation network index (GIPNI) was developed to assess the prognostic relevance of gene interaction perturbation network genes using a multi-step modeling strategy, according to a previous study 26. Firstly, the genes were calculated the meta-p values of GSE53624 and TCGA-ESCC cohorts based on univariate Cox p values of the two individual cohorts. Then, we adopted a two-stage strategy for machine-learning model construction. In the first stage, feature selection was performed using multiple algorithms, including StepCox, LASSO (Least Absolute Shrinkage and Selection Operator), random survival forest (RSF), and CoxBoost, to minimize the number of variables entering the final model and thereby reduce the risk of overfitting. In the second stage, the variables retained by each feature-selection method were subsequently entered into a panel of survival-learning algorithms, including StepCox, LASSO, RSF, elastic net (Enet), gradient boosting machine (GBM), partial least squares regression for Cox models (plsRcox), Ridge, survival support vector machine (survivalSVM), and SuperPC, to identify the optimal model. For example, a StepCox model constructed using variables preselected by RSF was denoted as “RSF + StepCox.” In addition, models using the same algorithm for both feature selection and model construction were also evaluated. Model performance was assessed using Harrell's concordance index (C-index) across all cohorts, including both the training and validation sets.

### Weighted gene correlation network analysis

Weighted gene correlation network analysis was applied to explore gene modules related to the gene interaction perturbation network index (GIPNI). The analysis was carried out following standard WGCNA procedures, starting with the selection of a soft-thresholding power that allowed the network to approximate a scale-free topology. This parameter was chosen empirically based on the overall network behavior rather than on a fixed cutoff. Pairwise correlations between genes were calculated and converted into a weighted adjacency matrix. This matrix was further processed to generate a topological overlap matrix for calculating the similarity of connection patterns among genes. To evaluate the relationship between gene modules and the phenotype, module eigengenes were correlated with GIPNI values. Modules showing relatively stronger correlations with GIPNI were retained and used for subsequent functional analyses.

### Functional enrichment, pathway activation, and immune infiltration evaluation

Metascape online platform (https://metascape.org/) was used to investigate the enriched pathways of the genes. This analysis integrated multiple comprehensive gene sets, including Gene Ontology, KEGG, and Reactome, etc. The quantification of pathway activation and the 28 kinds of immune cells were performed using the ssGSEA algorithm. Specifically, the pathway and the marker genes were defined by MsigDB database (www.gsea-msigdb.org) 27. The relationship between the quantified pathway activation scores and the GIPNI were evaluated through Spearman correlation analysis.

### Therapeutic response prediction

For predict half-maximal inhibitory concentration (IC50) values of common drugs, the oncoPredict R package was applied in the included ESCC cohorts 28. Immunotherapy clinical responses were evaluated by the Tumor Immune Dysfunction and Exclusion (TIDE) algorithm, which could be accessed at the online website (https://tide.dfci.harvard.edu/) 29.

### Performance evaluation of GIPNI and nomogram development

Harrell's concordance index was calculated for each dataset with functions provided in the pec package. To examine predictive accuracy over time in both internal and external cohorts, time-dependent area under the curve (AUC) analyses were performed by timeROC R package. Corresponding 95% confidence intervals were obtained through the built-in confidence interval procedures. Nomograms were constructed to visualize the predictive model, together with calibration plots to assess agreement between predicted and observed outcomes. These analyses were performed by the regplot and rms R packages. In addition, the clinical utility of the GIPNI was explored by decision curve analysis based on the ggDCA package.

### Statistical methodology

All statistical analyses were based on R software (version 4.2.3). Comparisons between two groups were performed using the limma package, the Wilcoxon rank-sum test, or Student's t test based on the respective datasets. For survival analysis, the cutoff values for GIPNI and selected genes were calculated according to the Log-rank test results provided by the survminer package. A two-sided p-value below 0.05 was considered statistically significant. For multiple comparisons, p-values were adjusted by the Benjamini-Hochberg method.

## Results

### Single-cell transcriptomic landscape of ESCC and identification of malignant epithelial cells

**Figure 1:**
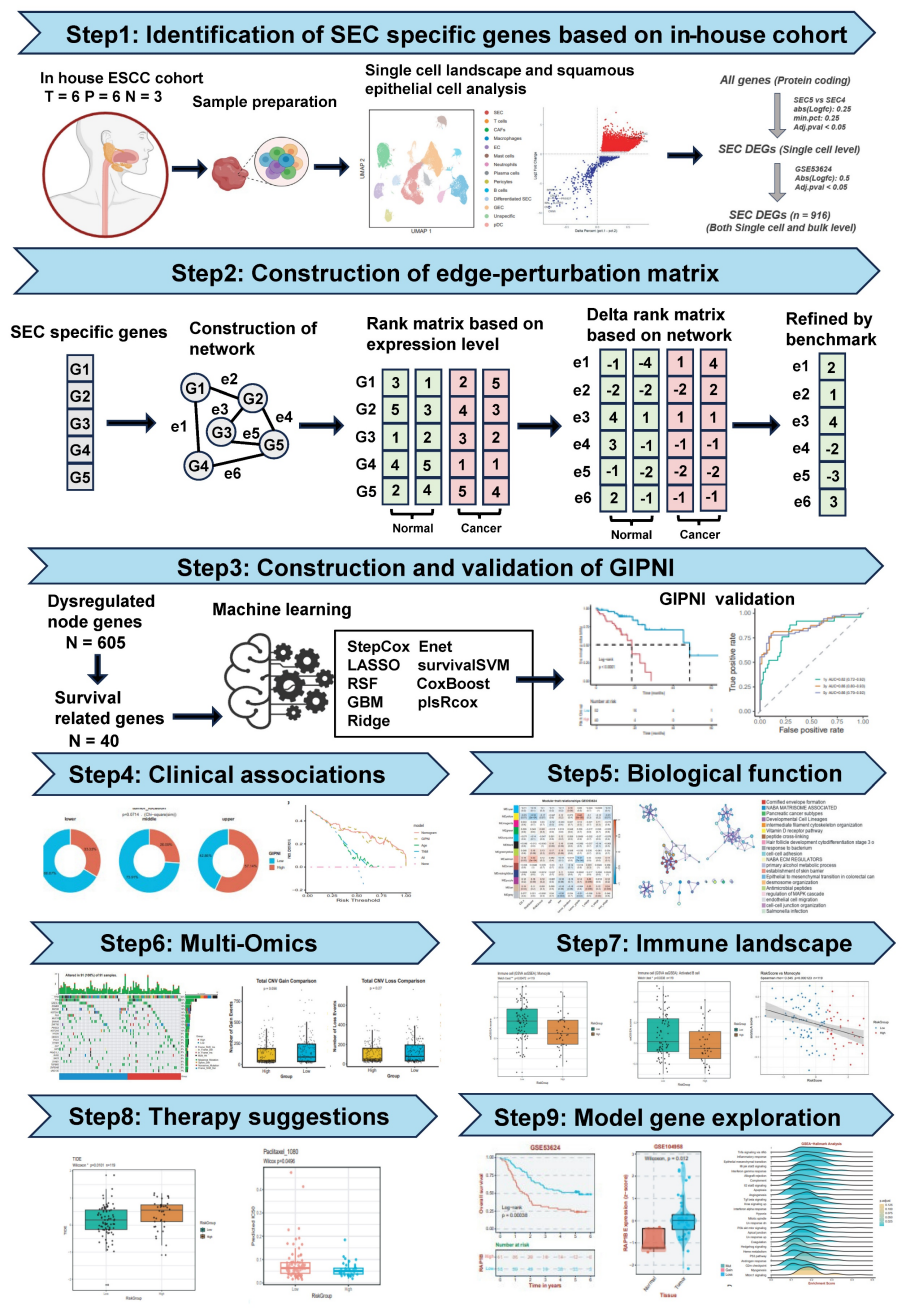
The overall workflow of this study.

**Figure 2:**
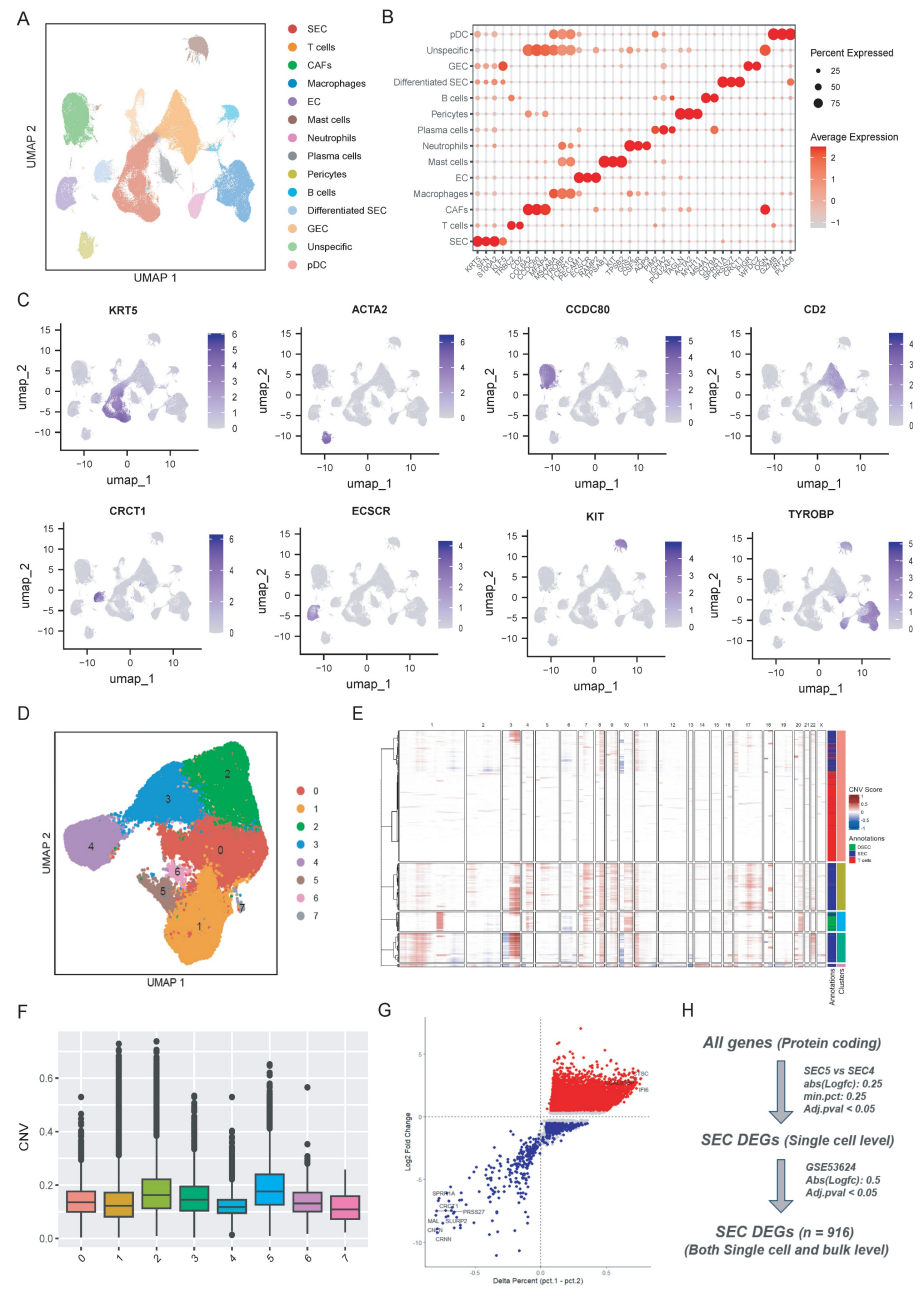
Single-cell transcriptomic profiling and identification of malignant epithelial cell subpopulations in ESCC. (A) UMAP visualization of 13 major cell lineages identified from the integrated ESCC single-cell dataset. (B-C) Dot plot and UMAP feature plots displaying the expression of canonical lineage-specific markers used for cluster annotation. (D) UMAP plot of the squamous epithelial cell (SEC) population. (E-F) Inference of chromosomal copy number variations (CNV) based on the fastCNV algorithm. (G) Volcano plot showing the differentially expressed genes (DEGs) between the malignant SEC5 and SEC4 clusters. (H) Schematic flowchart of the integrative strategy used to identify robust SEC-specific DEGs.

The flowchart of this study was displayed in Fig. 1. We collected 15 samples from the First Affiliated Hospital of Zhengzhou University, comprising 6 tumor tissues, 6 paratumoral tissues, and 3 normal tissues for single-cell RNA sequencing. After sequencing, the data was processed using the Seurat package. Quality control results were displayed in Supplementary Fig. 1A-D. Finally, 224,063 high-quality cells were remained to characterize the single cell landscape of the esophageal squamous cell carcinoma (ESCC). Then, we performed unsupervised clustering and UMAP visualization on the scRNA-seq dataset. As shown in Fig. 2A, we identified 13 distinct cell types, including squamous epithelial cells (SEC), T cells, CAFs, macrophages, endothelial cells (EC), mast cells, neutrophils, plasma cells, pericytes, B cells, differentiated SEC, glandular epithelial cells (GEC), and plasmacytoid dendritic cells (pDC). The defined cell clusters were marked by the typical cell markers (Fig. 2B and 2C), such as KRT5 and CRCT1 for SECs, ACTA2 for CAFs, CCDC80 for pericytes, CD2 for T cells, ECSCR for ECs, KIT for mast cells, and TYROBP for macrophages. Additional markers are visualized in Supplementary Fig. 2.

To further explore the heterogeneity squamous epithelial cells, the SEC and differentiated SEC population were selected and reclustered into 8 subpopulations (Fig. 2D). We then used the fastCNV algorithm to distinguish malignant squamous epithelial cells from the normal epithelial cells by estimating the large-scale chromosomal copy number variations (CNV) 23. As displayed in Fig. 2E and 2F, cluster 5 exhibited the highest CNV scores compared to other sub-clusters and cluster 4 showed the lowest CNV scores. Then as described in Fig. 2H, we performed single-cell level differentially analysis between cluster 5 and cluster 4 (Fig. 2G) with a threshold of |Log_2FC| > 0.25 and Adj.p < 0.05. These differential expression genes (DEGs) at single-cell level were then cross-validated with bulk transcriptomic data from the GSE53624 dataset (|Log_2FC| > 0.5 and Adj.p < 0.05). In total, 916 SEC-specific DEGs that were consistently dysregulated at both single-cell and bulk-transcriptome levels were identified by this integrative approach (Fig. 2H). The gene list is provided in Supplementary Table 1.

### Establishment of the gene interaction perturbation network and the molecular subtypes in ESCC

**Figure 3:**
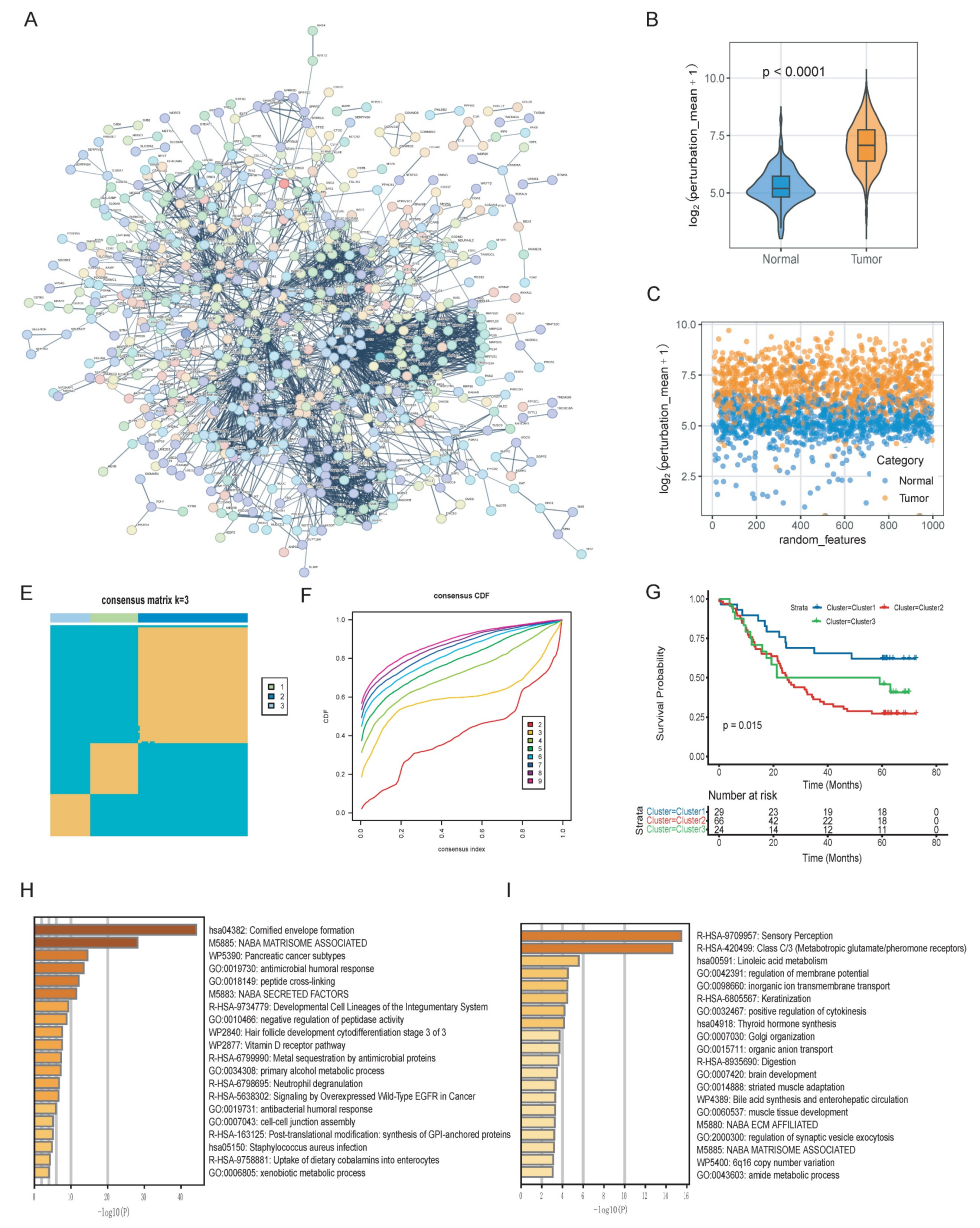
Construction of gene interaction perturbation network and molecular subtype stratification in ESCC. (A) Protein-protein interaction (PPI) network of malignant-associated DEGs. (B-C) Violin plot and scatter plot showing the distribution of the gene crosstalk perturbation mean (log_2) in tumor versus normal tissues, indicating significantly higher network instability in malignant samples. (E-F) Consensus clustering analysis of ESCC samples. The consensus matrix (E) and CDF curves (F) identified three stable molecular clusters (k = 3). (G) Kaplan-Meier curves demonstrating significantly different OS among the three identified molecular subtypes (p = 0.015). (H-I) Enrichment analysis of subtype-specific pathways. Bar plots represent the top enriched biological processes and signaling pathways for Cluster 1 (H) and Cluster 3 (I), respectively.

To further investigate the functional interplay among the previously identified 916 DEGs, we constructed a protein-protein interaction (PPI) network to reflect the gene interaction perturbations by performing String analysis (Fig. 3A) 30. This network comprised 914 nodes and 2,815 edges, with an average node degree of 6.16 (Supplementary Table 2). We first integrated normal esophageal squamous epithelial tissue data from the GTEx cohort and esophageal squamous cell carcinoma data from the TCGA-ESCC cohort. The PCA results of merged cohort were displayed in Supplementary Fig. 3A-3B. Then, as the method described by previous study 13, we established a robust benchmark for edge perturbation based on normal tissues (Fig. 1). Then, we calculated the edge perturbation matrix for normal and tumor tissues. As shown in Fig. 3B, tumor tissues displayed significantly higher levels of network perturbation compared to the normal tissues (p < 0.0001). The randomly selected specific interaction edges to visualize the divergence, result demonstrated a significant distinction, which suggested the edge perturbation matrix was well constructed (Fig. 3C).

To investigate the molecular features of these network dynamics, we performed the consensus clustering by the edge perturbation matrix via the ConsensusClusterPlus package 31. The consensus matrix and cumulative distribution function (CDF) curves indicated that three was the optimal number of clusters (Fig. 3E and 3F). Survival analysis revealed significant prognostic heterogeneity among these subgroups (p = 0.015). Among them, Cluster 3 exhibited the most favorable survival outcomes, while Cluster 2 was associated with the poorest prognosis (Fig. 3G). Functional pathway enrichment results showed the divergent biological mechanisms driving these subtypes. Of which, Cluster 1 was enriched in cornified envelope formation, matrisome-associated factors, and antimicrobial humoral responses pathways (Fig. 3H). Cluster 3 showed distinct enrichment biological processing in sensory perception, linoleic acid metabolism, and ion transmembrane transport (Fig. 3I).

### Development and validation of the gene interaction perturbation network index (GIPNI) in ESCC

To further enhance the clinical applications and interpretability of the constructed gene interaction perturbation network, we identified the interaction edges that were significantly dysregulated between the tumor and normal tissues. Then, we extracted the edge-related node genes (Supplementary Table 1). These genes were regarded as represented not only the dysregulated expression both in the single-cell and bulk levels but also the intrinsic characteristics of the gene interaction perturbation network. Then, we integrated the prognostic data from the TCGA-ESCC and GSE53624 datasets and further selected 40 survival-related genes, which were significantly associated with OS (Supplementary Table 3). The whole workflow was shown in Fig. 1. After that, the prognosis genes were generated into a machine learning framework which had 75 algorithm combinations including Stepwise Cox, Lasso, plsRcox, GBM, Enet, survivalSVM, SuperPC, CoxBoost, Ridge, and Random Survival Forest. Results showed the Stepwise Cox-based models exhibited the best performance across the training and validation cohorts. Specifically, the StepCox model achieved a C-index exceeding 0.77 in the training set (GSE53624), while maintaining C-indices of 0.65 in TCGA-ESCC and 0.59 in GSE53622. The final model, termed the Gene Interaction Perturbation Network Index (GIPNI), demonstrated robust predictive accuracy and generalizability with a mean C-index of 0.67 across all cohorts.

**Figure 4:**
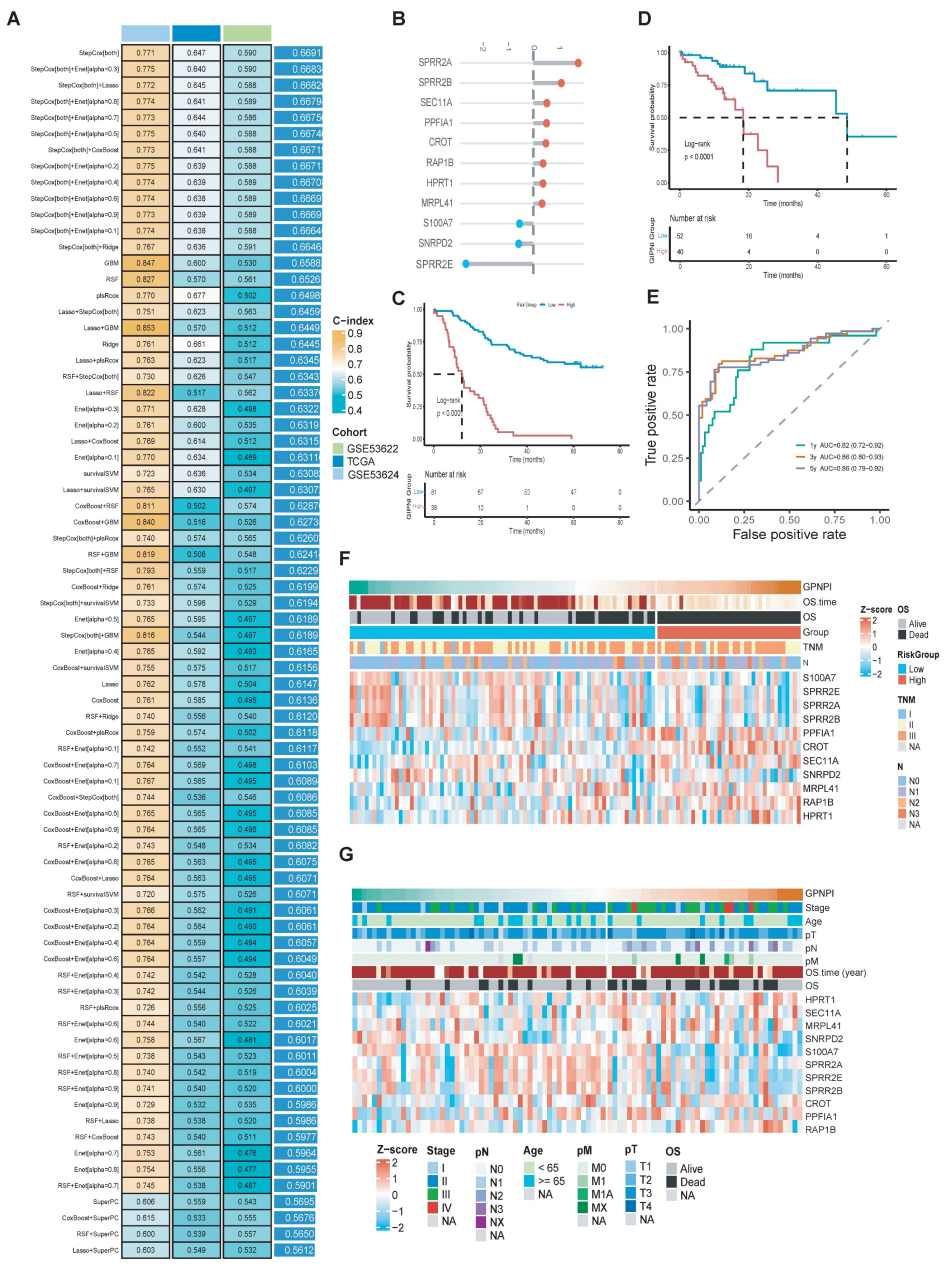
Construction and multi-cohort validation of the gene interaction perturbation network index (GIPNI) in ESCC. (A) Performance comparison of various machine learning algorithm combinations, including Stepwise Cox, Lasso, and RSF, based on their C-index values across multiple datasets. (B) The coefficient value of 11 model gens. (C) KM curve of OS for high- and low-GIPNI groups in the GSE53624 cohort. (D) KM curve of OS in the TCGA-ESCC cohort. (E) Time-dependent ROC curves for 1-, 3-, and 5-year survival predictions in the GSE53624 cohort, showing corresponding AUC values. (F-G) Heatmaps and distribution plots illustrating GIPNI scores, survival status, clinical stages, and the expression patterns of the 11 signature genes in the GSE53624 (F) and TCGA-ESCC (G) cohorts.

GIPNI was consisted of 11 genes including SPRR2A, SPRR2B, SPRR2E, SEC11A, PPFIA1, CROT, RAP1B, HPRT1, MRPL41, S100A7, and SNRPD2. The coefficients of these genes are displayed in Fig. 4B. Then, we performed the Kaplan-Meier analysis to evaluate the risk stratification ability in ESCC. Results showed the high-risk patients had the worse prognosis in TCGA and GSE53624 cohorts (Fig. 4C and 4D; all p values < 0.0001). The area under the curve (AUC) values were 0.82 at 1 year, 0.85 at 3 years, and 0.86 at 5 years OS in the GSE53624 cohort (Fig. 4E). Then, we used heatmap for visualization of the GIPNI score patient status. Results demonstrated that increased GIPNI scores were positively correlated with higher mortality rates and more advanced clinical stages, respectively in the GSE53624 (Fig. 4F) and TCGA (Fig. 4G) cohort.

### Clinical associations and independent prognostic value of the GIPNI

**Figure 5:**
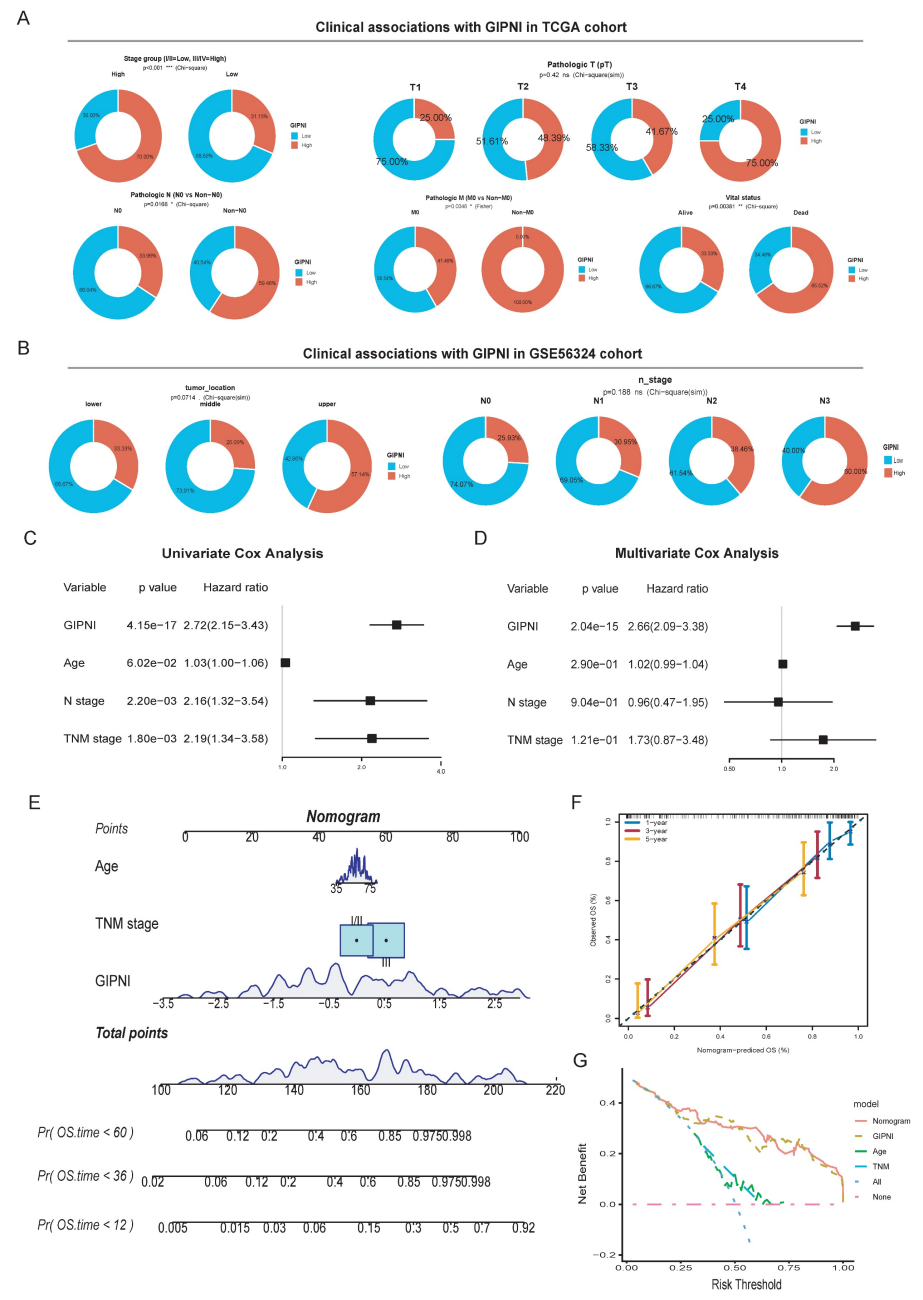
Clinical significance and independent prognostic validation of the GIPNI. (A) Distribution of clinico-pathological features (Stage, Pathologic N, T, and M stages) stratified by GIPNI in the TCGA cohort. (B) Association of GIPNI scores with clinical parameters, including tumor location, vital status, and N-stage in the GSE53624 cohort. (C-D) Univariate and multivariate Cox regression analyses results of GIPNI in GSE53624 cohort. (E) Nomogram integrated with the GIPNI and key clinical features for predicting OS. (F) Calibration curves showing the alignment between nomogram-predicted and actual survival probabilities. (G) DCA comparing the clinical net benefit of the GIPNI, the nomogram, and traditional clinical traits, highlighting the robust predictive performance of the GIPNI.

We analyzed the associations of GIPNI and various clinical pathological features. In the TCGA-ESCC cohort, the high-GIPNI ESCCs had a higher proportion of clinical stages III-IV compared to the low- GIPNI ESCCs (Fig. 5A). Patients with lymph node metastasis (non-N0) or distant metastasis (non-M0) tended to have higher GIPNI scores, but the difference in pathologic T-stage was smaller (Fig. 5A). Similar clinical associations were observed in the GSE53624 cohort. GIPNI scores were significantly higher in patients with advanced N-stages (N2-N3) and in patients who died, showing its link to tumor aggressiveness (Fig. 5B). In contrast, no significant difference was found between GIPNI and demographic or lifestyle factors, including sex, tobacco use, alcohol use, and age (P > 0.05) (Supplementary Fig. 4A-E).

To determine whether the GIPNI was an independent prognostic index, we performed univariate and multivariate Cox regression analyses. Results indicated that the GIPNI was significantly associated with overall survival by univariate analysis (Hazard Ratio [HR] = 2.72, 95% CI: 2.15-3.43, p < 0.001) (Fig. 5C). By adjusting for other ESCCs clinical variables including age and pathologic stage, multivariate Cox analysis confirmed that the GIPNI was the only independent predictor indicating the poor prognosis (HR = 2.66, 95% CI: 2.09-3.38, p < 0.001) (Fig. 5D). For clinical application, we integrated the GIPNI with important clinical factors to construct a comprehensive nomogram (Fig. 5E). The calibration curves of different survival periods indicated the model's high reliability (Fig. 5F). Finally, to evaluate the model's clinical net benefit, we performed the decision curve analysis (DCA). Notably, while the combined nomogram showed the highest benefit, the GIPNI alone demonstrated a predictive power comparable to the integrated model and significantly outperformed individual clinical features including TNM stage (Fig. 5G).

### Genomic alteration landscape of GIPNI subgroups

**Figure 6:**
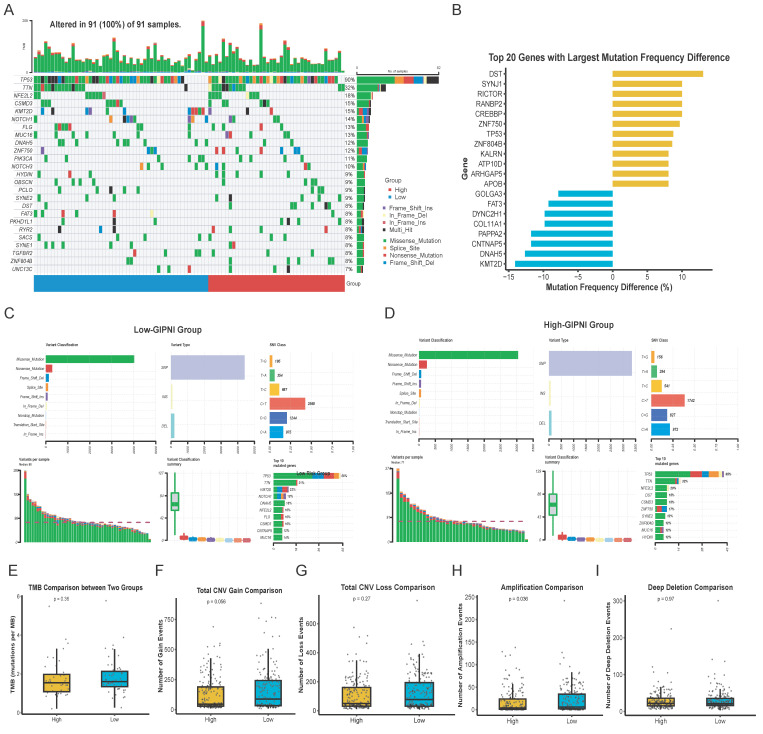
Genomic landscape and somatic mutation profiles stratified by GIPNI risk groups. (A) Oncoplot visualizing the somatic mutations of the most frequently altered genes in the TCGA-ESCC cohort. (B) Comparison of the top 20 genes with the largest mutation frequency differences between high- and low-risk GIPNI groups. (C-D) Detailed mutational profiles for the low-risk (C) and high-risk (D) groups, illustrating the distribution of mutation types, including missense, nonsense, and frame-shift alterations. (E-I) Statistical analysis comparing tumor mutation burden (TMB) (E), total CNV gain (F), total CNV loss (G), amplification (H), and deep deletion (I) between high- and low-GIPNI patients, with p-values indicated above each plot.

We analyzed somatic mutations and copy number variations (CNV) in the TCGA-ESCC cohort to investigate the genomic alterations of the GIPNI. Results revealed that TP53, TTN, KMT2D, and NOTCH1 were among the most frequently mutated genes in all samples (Fig. 6A). By comparing the mutational frequencies between the two GIPNI groups, we calculated the top 20 genes with the most significant deviations (Fig. 6B). High-GIPNI patients showed a markedly higher prevalence of gene mutations such as DST, SYNJ1, RICTOR, and CREBBP compared to the low-GIPNI group (Fig. 6B, 6D). The separate oncoplots (waterfall plots) for the two groups are presented in Supplementary Fig. 5A-B, while the genes which had the most significant differences of mutation frequency are summarized in Supplementary Fig. 5C. Besides, the summary of mutation in high- and low- GIPNI was displayed in Fig. 6C and 6D. Although the overall tumor mutational burden (TMB) showed no statistical difference (Fig. 6E), the low-GIPNI group exhibited a higher trend in CNV gain (Fig. 6F; P = 0.056) and a significantly higher frequency of amplification events (Fig. 6H; P = 0.036). No significant differences were exhibited in different GIPNI groups regarding CNV loss or deep deletion (Fig. 6F and Fig. 6I).

### Integrative analysis of GIPNI-Associated biological pathways and functional modules via WGCNA

**Figure 7:**
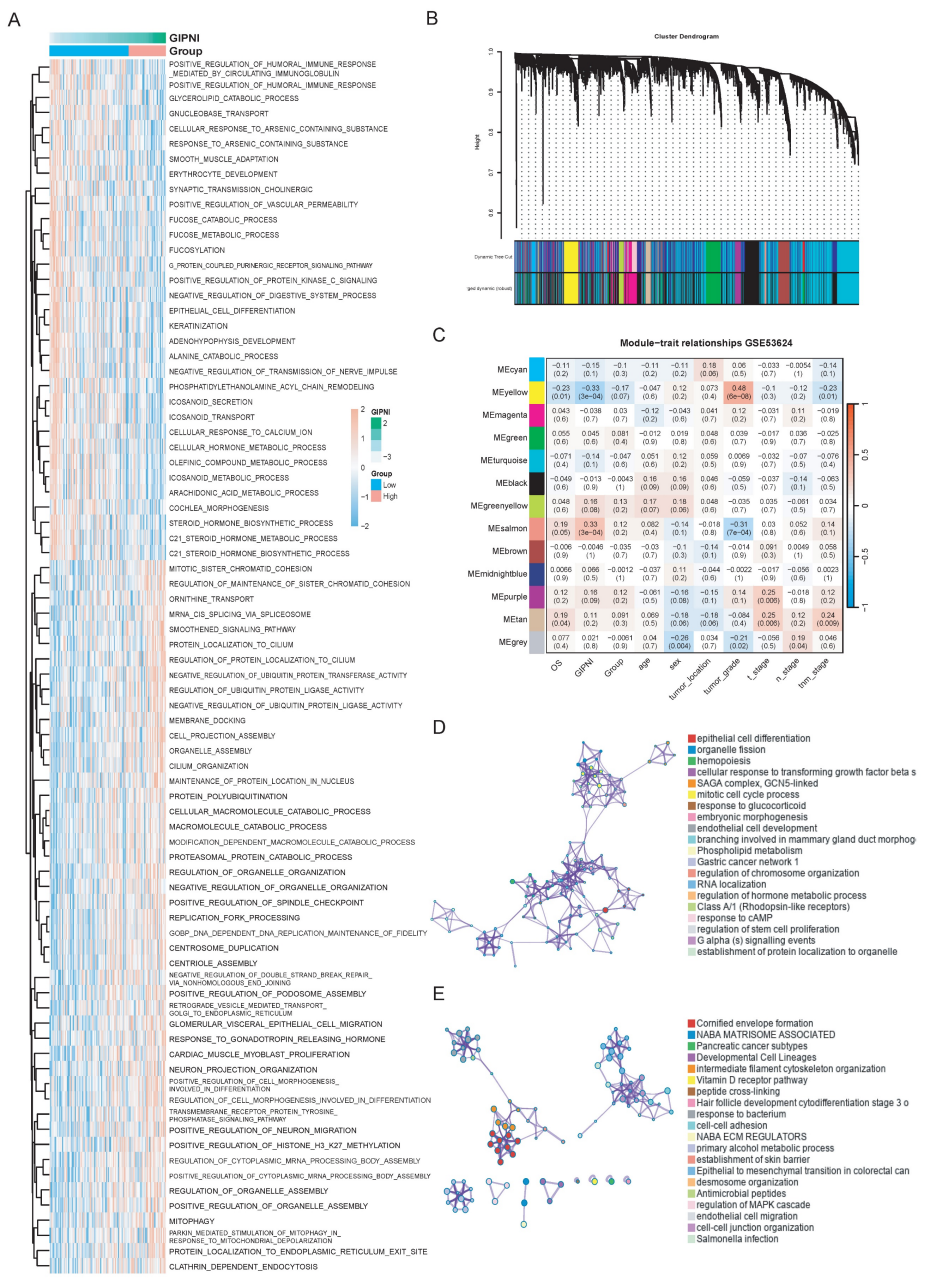
Characterization of GIPNI-related biological pathways and functional modules. (A) Heatmap showing the correlation between GIPNI scores and GO biological processes (BP) analyzed by ssGSEA. (B) Cluster dendrogram of based on WGCNA results. (C) Module-trait relationship heatmap visualizing the correlation between gene modules and clinical traits (GIPNI score, age, gender, stage, and survival status). (D) Bar plot illustrating the top enriched GO terms for genes in the Salmon module, which is positively correlated with the GIPNI. (E) Bar plot illustrating the top enriched GO terms for genes in the yellow module, which is negatively correlated with the GIPNI.

To further elucidate the biological mechanisms by which the gene interaction perturbation network index (GIPNI) modulates ESCC progression, we first employed the ssGSEA algorithm to quantify the activity of biological pathways across the TCGA-ESCC cohort. Fig. 7A showed the GOBP (GO biological processes) pathways which were significantly associated to the GIPNI. High GIPNI scores were significant positively correlated with the positive regulation of humoral immune response, cell morphogenesis, and neuron migration pathways. The negative correlation pathways including keratinization and epithelial cell differentiation which were essential for maintaining epithelial homeostasis (Fig. 7A). In support of this notion, a recent study demonstrated that ESCCs with an activated epithelial keratinization program exhibited a more favorable prognosis 10.

To identify the GIPNI related gene modules, the weighted gene co-expression network analysis (WGCNA) was performed in GSE53624 cohort. The hierarchical clustering of samples after outlier removal was shown in Supplementary Fig. 6A. The selection of the soft-thresholding power was visualized in Supplementary Fig. 6B-C. All genes were clustered into distinct co-expression modules, which were displayed in the dendrogram (Fig. 7B). We then constructed a module-trait relationship heatmap to correlate the module genes with clinical characteristics and GIPNI (Fig. 7C). Among them, the Salmon module showed the most significant positive correlation with the GIPNI (r = 0.58, p = 1e-08), while the yellow module displayed a robust negative correlation (r = -0.52, p = 2e-07). Scatter plots showed the module membership (MM) versus gene significance (GS) (Supplementary Fig. 6D-E). Interestingly, the two related modules also exhibited consistent correlation trends with clinical features such as stage and survival status, suggesting their roles in the malignant progression of ESCC (Fig. 7C). To analyze the biological pathways of genes within the two modules, we performed functional enrichment analysis by Metascape 32. The salmon module genes were significantly enriched in organelle assembly, positive regulation of humoral immunity, mitophagy, and cell morphogenesis pathways (Fig. 7D). The yellow module genes functioned in pathways including keratinization, establishment of the skin barrier, arachidonic acid metabolism, and steroid hormone biosynthesis (Fig. 7E). Above results demonstrated that the GIPNI served as a comprehensive indicator of the molecular features in ESCC. Mechanistically, an increased GIPNI score was associated with the loss of epithelial differentiation features and the acquisition of immune remodeling and invasive phenotypes, which may partly explain the poorer prognosis observed in ESCC patients with high GIPNI.

### Application of GIPNI in predicting immunotherapy response and chemotherapeutic sensitivity

**Figure 8:**
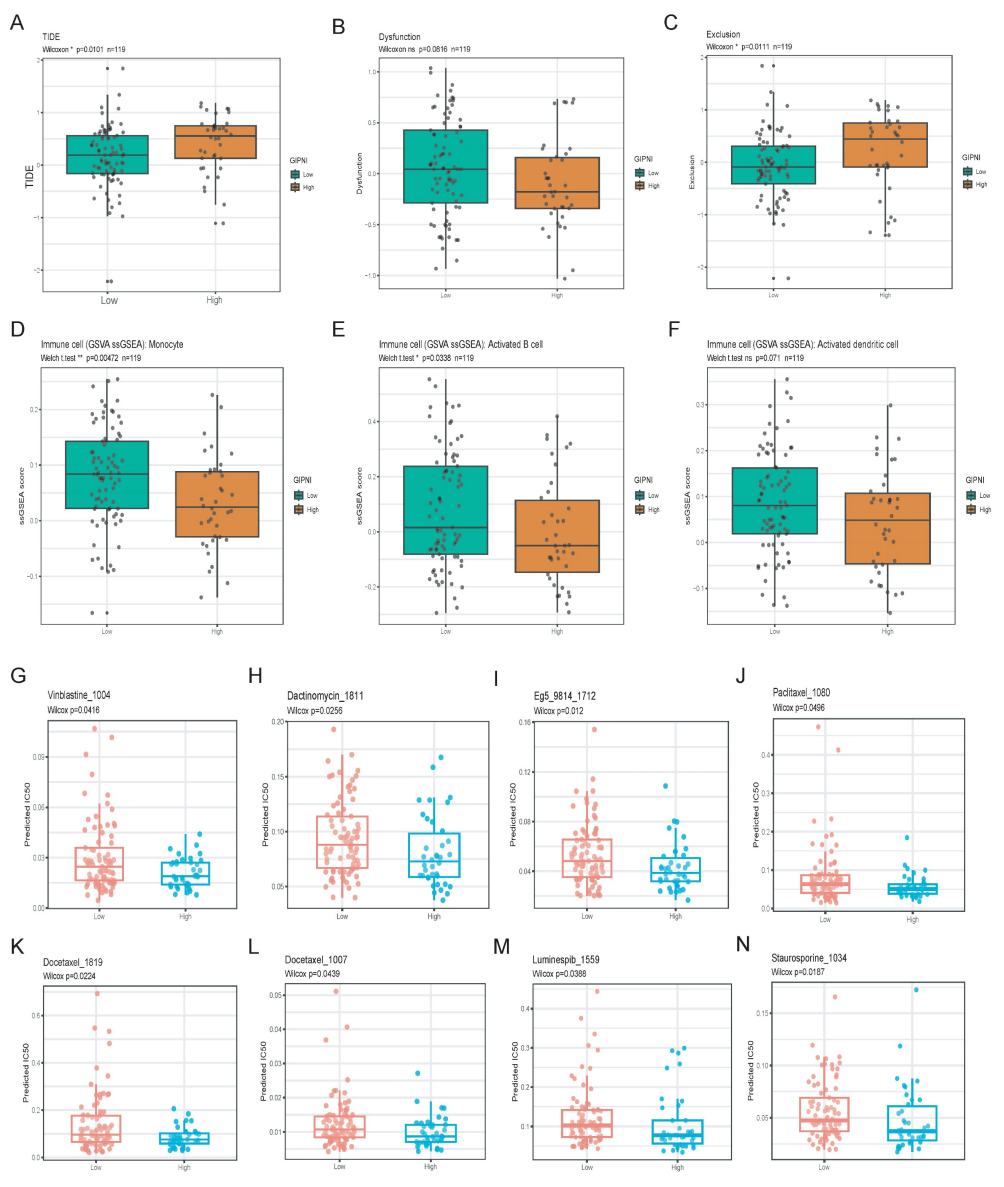
Evaluation of therapeutic response and immune infiltration across GIPNI risk groups. (A-C) Boxplots comparing TIDE, T-cell dysfunction, and exclusion scores between high- and low-GIPNI groups. (D-F) Distribution of monocytes (D), activated B cells (E), activated dendritic cell (F) between high- and low-GIPNI groups. (G-N) Predicted IC50 values results based on oncoPredict.

To evaluate the clinical application of treatment response based on GIPNI, we investigated its association with immunotherapy response by TIDE algorithm 29. Compared to those in the high-GIPNI ESCCs, the low-GIPNI ESCCs exhibited significantly lower TIDE scores (Fig. 8A; p = 0.010), which indicated a higher immune checkpoint inhibitors response. This result was further supported by the T-cell dysfunction and exclusion scores (Fig. 8B and 8C). Thus, we regarded high-GIPNI ESCC as a "cold" or immune-evasive microenvironment compared to the low-GIPNI ESCC. To validate these results, we performed immune infiltration analysis including 28 kinds of immune cell types. Consistent with the TIDE predictions, we observed the significant immune cell infiltration differences in two GIPNI groups. Specifically, the low-GIPNI ESCC showed significantly higher infiltration of monocytes (Fig. 8D; p = 0.0047), activated B cells (Fig. 8E; p = 0.0338), and activated dendritic cell (Fig. 8F; p = 0.071). Among these immune components, dendritic cell (DC) activation is essential for the initiation of adaptive immune responses. Previous studies have suggested that impaired or exhausted DCs constitute an important contributor to immune evasion in ESCC 4. Other immune cell infiltration results were provided in Supplementary Fig. 7. All above results showed the antigen presentation and anti-tumor immune response were more active, which indicated the more sensitivity to immunotherapy in low-GIPNI ESCC.

For exploration of available drugs in high-GIPNI ESCC, we predicted the half-maximal inhibitory concentration (IC50) for common FDA-approved drugs including conventional and targeted chemotherapeutic agents based on the oncoPredict R package in GSE53624 cohort. Among these available drugs, high-GIPNI ESCC showed more sensitivity to taxanes including docetaxel (Fig. 8K and 8L; p < 0.05), and vinca alkaloids such as vinblastine (Fig. 8G; p = 0.0416). In clinical practice, docetaxel was applied for advanced ESCC, which was often used in combination with cisplatin or as a second-line monotherapy. Furthermore, high-GIPNI patients showed more sensitivity to dactinomycin (Fig. 8H; p = 0.0256), Eg5 inhibitors (Fig. 8I; p = 0.012), and luminespib (Fig. 8M; p = 0.0388). Of which, luminespib was an HSP90 inhibitor. HSP90 family genes were widespread significantly overexpressed in tumor tissues in almost all validated ESCC cohorts (Supplementary Fig. 8), similar to previous studies 33, 34. These results provided therapeutic directions for high-GIPNI ESCC. In summary, the low-GIPNI ESCC was ideally suited for immunotherapy, and the high-GIPNI ESCC might derive significant clinical benefit from personalized chemotherapy.

### Multi-omics characterization and external validation of the model genes

**Figure 9:**
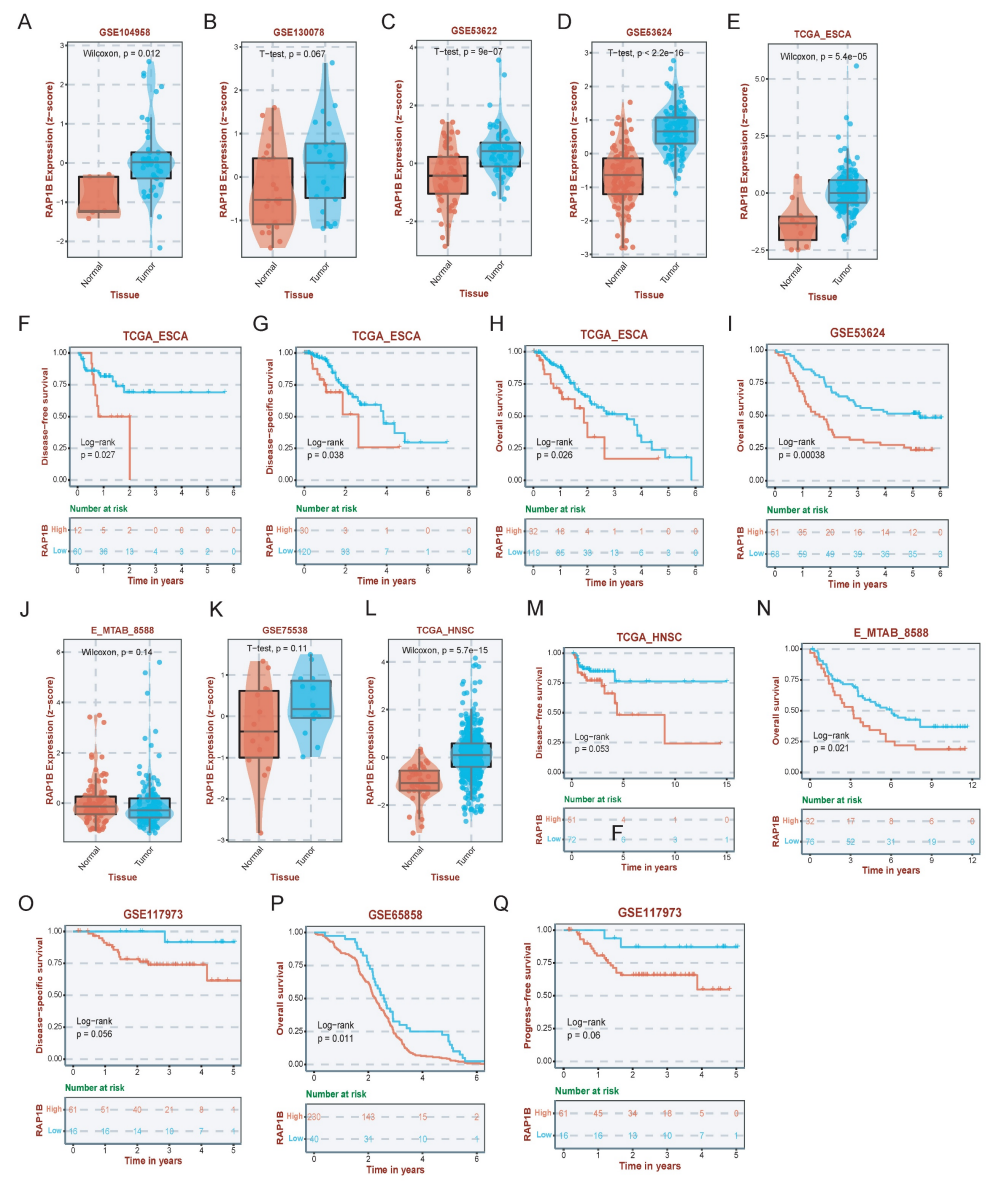
Expression and prognostic validation of RAP1B in ESCC and HNSC. (A-E) Expression levels of RAP1B in tumor and normal tissues across various ESCC datasets (GSE104958, GSE130078, GSE53622, GSE53624, and TCGA-ESCA). (F-I) KM curves displaying the relationship of RAP1B with OS, DFS, and DSS in ESCC cohorts. (J-L) Validation of RAP1B overexpression in HNSC tumor tissues across independent cohorts (GSE75538, E-MTAB-8588, and TCGA). (M-Q) Survival analysis demonstrating the prognostic impact of RAP1B on OS and DSS in HNSC patients.

To further investigate the potential drug targets of GIPNI model genes, we performed a systematic validation of each gene. Results showed that nearly all identified genes were significantly dysregulated across multiple independent ESCC cohorts (Supplementary Fig 9). Of which, RAP1B, a member of the Ras related protein family known to regulate cell adhesion and migration as well as integrin mediated signaling, was prioritized for detailed characterization due to its important role in tumor progression 35. In ESCC, RAP1B was significantly upregulated in ESCC tumor tissues across several independent datasets, including GSE104958, GSE130078, GSE53622, GSE53624, and the TCGA-ESCA cohort (Fig. 9A-E). Survival analysis further showed that high RAP1B expression leaded to the worse overall survival (OS), disease-free survival (DFS), and disease-specific survival (DSS) in ESCC patients (Fig. 9F-I). Then, we used survival datasets from HNSC, another kind of squamous cell carcinomas, to evaluate the prediction ability of RAP1B. Interestingly, in HNSC cohorts including GSE75538 and E-MTAB-8588, RAP1B was similarly overexpressed in malignant tissues (Fig. 9J-L) and served as a robust predictor of poor clinical outcomes (Fig. 9M-Q).

**Figure 10:**
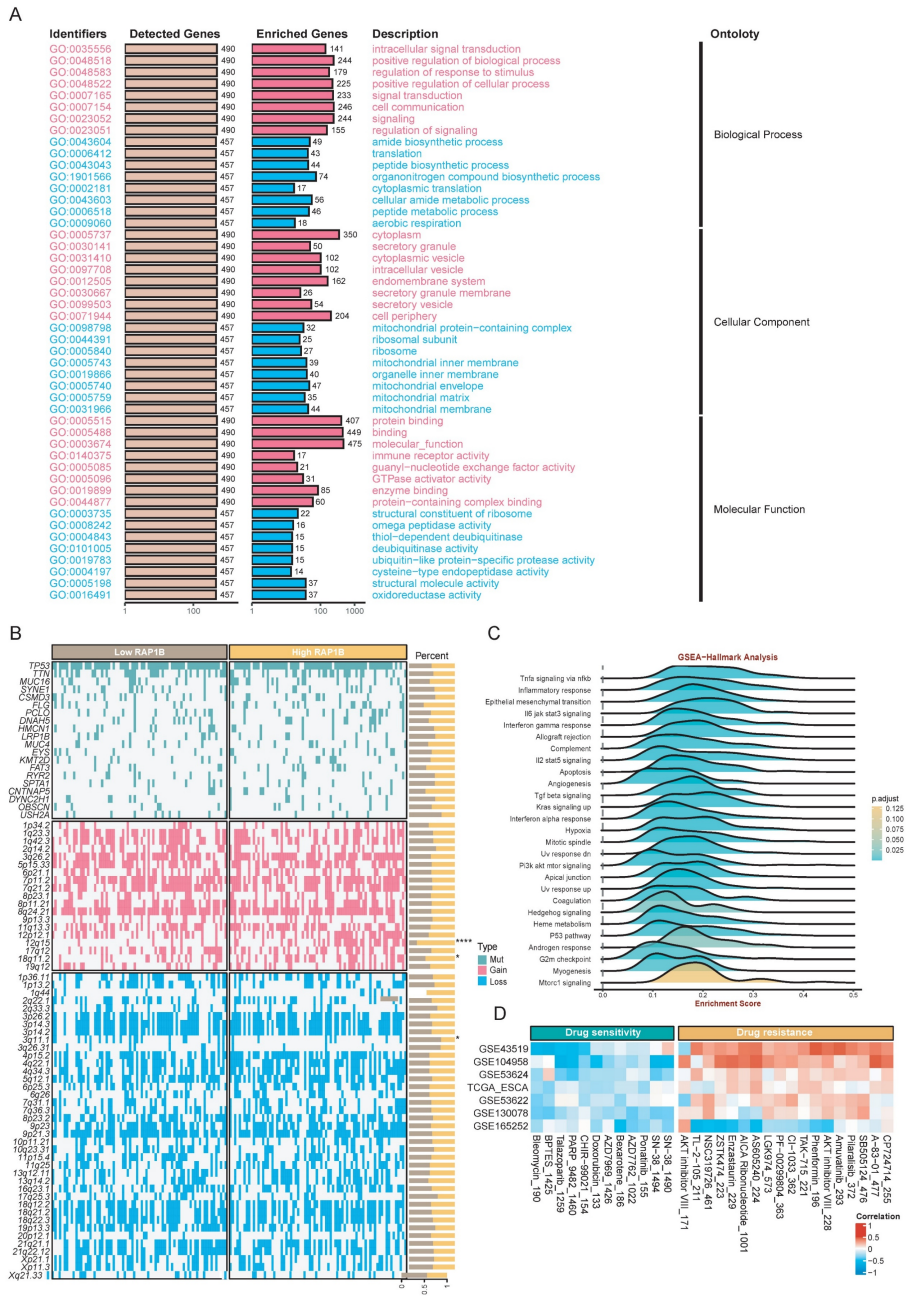
Multi-omics landscape and therapeutic vulnerabilities associated with RAP1B. (A) GO enrichment analysis showing the biological processes associated with RAP1B-related genes, emphasizing signal transduction and cell communication. (B) Somatic mutation and CNA landscape stratified by RAP1B expression levels. (C) GSEA-Hallmark pathways enriched in high-RAP1B samples. (D) Correlation between RAP1B expression and predicted drug sensitivity (IC50).

We then performed multi-omics analyses to investigate the RAP1B molecular mechanisms. Functional enrichment results showed RAP1B-related genes were enriched in intracellular signal transduction, cell communication, and amide biosynthetic processes (Fig. 10A). As for tumor-hallmark pathways, GSEA-Hallmark results showed that RAP1B was associated with hypoxia, epithelial mesenchymal transition, and TNF-alpha signaling via NF-kappa B (Fig. 10C). Genomic analysis showed RAP1B expression was associated with copy number gains at 12q15 and 18q11.2 and chromosomal loss at 3q11.1 (Fig 10B). Finally, we explored the therapeutic implications of RAP1B expression by predicting drug sensitivity (IC50). Results displayed several compounds with significant correlations between their estimated IC50 values and RAP1B expression levels (Fig. 10D), which might improve the treatment of high-RAP1B ESCCs.

## Discussion

Esophageal cancer presents high incidence and mortality rates worldwide, with squamous cell carcinoma being its primary histological form 2, 36, 37. Studying this tumor systematically across different levels can help clinicians evaluate patient prognosis and determine suitable treatments 11, 38-40. Using gene interaction network information may provide new insights into esophageal cancer, compared to static analysis of individual molecules 13. In this study, we developed a prognostic index for ESCC termed as gene interaction perturbation network index (GIPNI) by integrating malignant epithelial cell specific transcriptomic features with gene interaction perturbation network analysis. This strategy enabled robust risk stratification across multiple independent cohorts and demonstrated favorable prognostic performance compared with traditional TNM stage 37.

In this study, multi-omics data was analyzed to investigate the biological difference in high- and low- GINPI ESCCs. At the genomic level, low-GINP tumors showed low-GIPNI group exhibited a higher trend in CNV gain and a significantly higher frequency of amplification events, suggested extensive differences at the genomic level between the two groups. Gao et al, reported the CNV events were strongly associated with the prognosis in ESCC 10. Functional enrichment analyses indicated that high-GIPNI ESCC had the overactivated mitotic cell cycle and the suppressed keratinization together with the epithelial cell differentiation pathways. Notably, keratinization has recently been reported to be associated with a favorable prognosis and reduced post-chemotherapy recurrence in ESCC 10. Cell cycle related pathways could promote the progression of ESCC, and had shown good effects by blocking cell cycle related proteins 41, 42. These pathways were significantly enriched in different algorithms such as WGCNA and ssGSEA, revealing the reason of poor prognosis of high GIPNI ESCC.

Immunotherapy has been used as an important treatment for ESCC, especially for advanced ESCC. The latest study showed that the combined therapy of tiragolumab with atezolizumab and chemotherapy could significantly improve the antitumor efficacy compared to patients under chemotherapy regimens with or without atezolizumab treatments 43. However, a multicenter study from China reported that in the ESCC cohort with overall neoadjuvant chemoimmunotherapy, adjuvant immunotherapy did not show a survival benefit, but improved the OS of patients with residual tumors 44. The above highlights the key to identify the immune sensitive population in esophageal cancer. In this study, we found that low-GINPI ESCC overall presented a relatively activated immune microenvironment, including monocytes, DC cells, etc., and TIDE score also showed the benefits of immunotherapy for this population. High-GINPI ESCC shows sensitivity to docetaxe, dactinomycin, paclitaxel, and other chemotherapeutic drugs, some of which have been used in clinical practice 45, 46. In addition, High-GINPI ESCCS also exhibit sensitivity to HSP90 inhibitors such as luminespib, of which HSP90 is considered as a highly potential target for ESCCS 46, 47.

The GIPNI consisted of 11 genes, including SPRR2A, SPRR2B, SEC11A, PPFIA1, CROT, RAP1B, HPRT1, MRPL41, S100A7, SNRPD2, and SPRR2E. Some of these genes such as SPRR2A, SPRR2B, SPRR2E, and S100A7 had been reported in ESCC 48-51. Our results showed that the remaining model genes were also strongly associated with ESCC and were validated by multiple independent cohorts. Of which, RAP1B showed sustained high expression in both ESCC and HNSC cohorts, and had clear prognostic significance. The results suggested that RAP1B could serve as a representative biomarker in a broader perturbation network. Subsequent enrichment analysis revealed that RAP1B was associated with pathways such as EMT and hypoxia, which had been reported in ESCC 52, 53.

This study has several limitations. Firstly, while the single-cell RNA profile was collected exclusively from our team, integrating additional large-scale public scRNA-seq datasets might improve the identification of SEC dysregulated genes. Secondly, while immune response and drug sensitivity were predicted via computational tools, these findings necessitate further clinical validation to confirm their actual therapeutic relevance in ESCC patients. Thirdly, the GIPNI was constructed based on retrospective datasets and lacked validation in prospective ESCC cohorts. Finally, additional *in vitro* and *in vivo* experiments should be performed to further elucidate the biological functions of the genes included in the GIPNI model.

## Conclusion

By integrating in-house single-cell transcriptomics with gene interaction networks, we established a robust gene interaction perturbation network index (GIPNI) for ESCC risk stratification and treatment selection. The high- and low- GIPNI ESCCs were had the distinct prognosis, activated biological pathways, immunotherapy and chemotherapy response, which could help clinicians in decision-making.

## Supplementary Material

Supplementary figures.

Supplementary table 1.

Supplementary table 2.

Supplementary table 3.

## Data Availability

TCGA-ESCC and TCGA-HNSC datasets can be downloaded from the TCGA website (https://portal.gdc.cancer.gov/). All GEO datasets in this study can be downloaded from https://www.ncbi.nlm.nih.gov/geo/. The in-house single-cell sequencing data of ESCC can be accessed from the corresponding author upon reasonable request.

## Abbreviations

ESCC: Esophageal squamous cell carcinoma
GIPNI: Gene interaction perturbation network index
CAR-T: Chimeric Antigen Receptor T-Cell Immunotherapy
PCA: Principal component analysis
UMAP: Uniform Manifold Approximation and Projection
CNV: Copy number variation
LASSO: Least Absolute Shrinkage and Selection Operator
RSF: Random survival forest
Enet: Elastic net
GBM: Gradient boosting machine
plsRcox: Partial least squares regression for Cox models
survivalSVM: survival support vector machine
WGCNA: Weighted gene correlation network analysis
IC50: Half-maximal inhibitory concentration
TIDE: Tumor Immune Dysfunction and Exclusion
SEC: Squamous epithelial cells
DEGs: Differential expression genes
PPI: Protein-protein interaction
