# Supplementary material for: A squamous epithelial gene interaction perturbation network index for risk stratification in esophageal squamous cell carcinoma
Source: J Cancer. 2026 Apr 8;17(4):905–24. doi: 10.7150/jca.132865 (PMC13105158; doi:10.7150/jca.132865)
Supplement: Supplementary file 1 — Supplementary figures. [file jcav17p0905s1.pdf]

Supplementary Materials

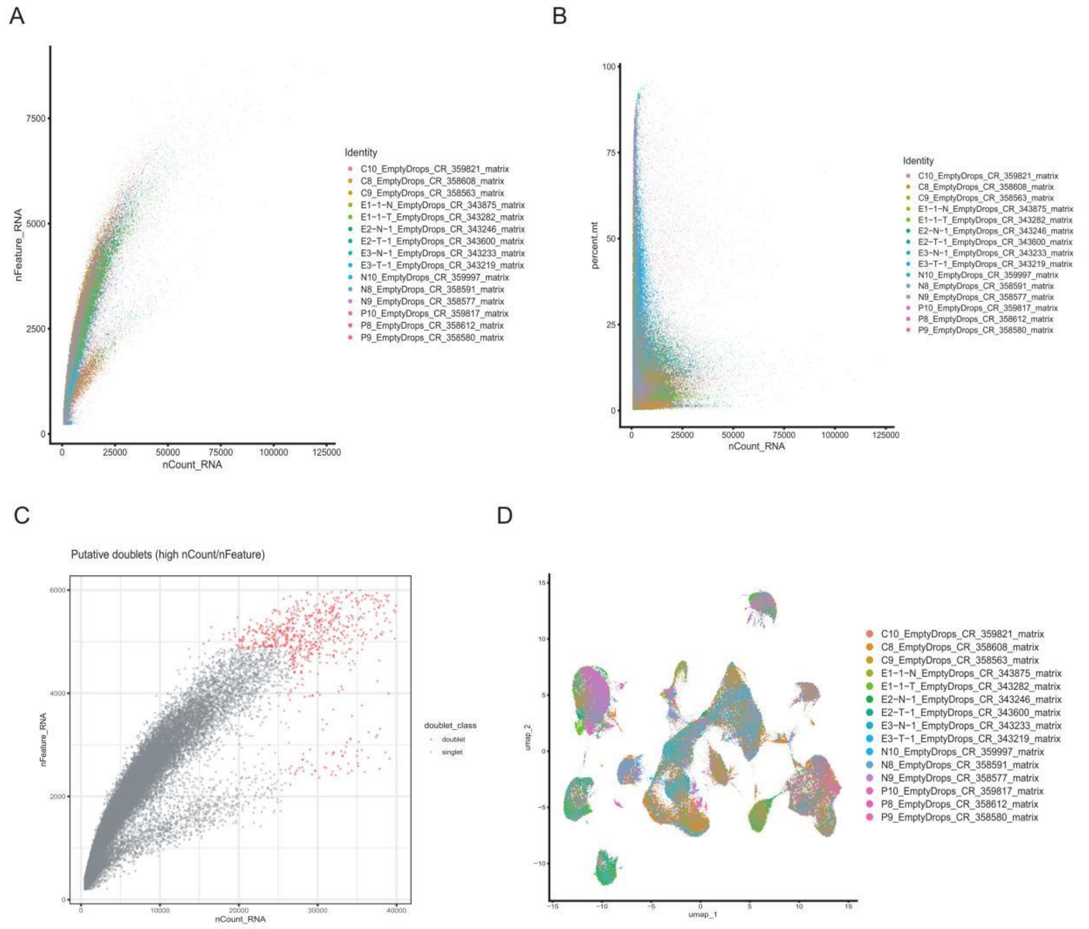

Figure S1. Quality control results of single cell analysis. (A) Scatter plot illustrating the positive correlation between the number of detected genes (nFeature_RNA) and the total number of unique molecular identifiers (nCount_RNA) per cell, color-coded by sample identity. (B) Distribution of the percentage of mitochondrial reads (percent.mt) relative to the total nCount_RNA. (C) Scatter plot highlighting putative doublets (red dots) identified based on unusually high nCount and nFeature ratios. (D) UMAP plot showing the successful integration of all samples. The overlapping distribution of different sample identities (represented by various colors) indicates the effective removal of batch effects.

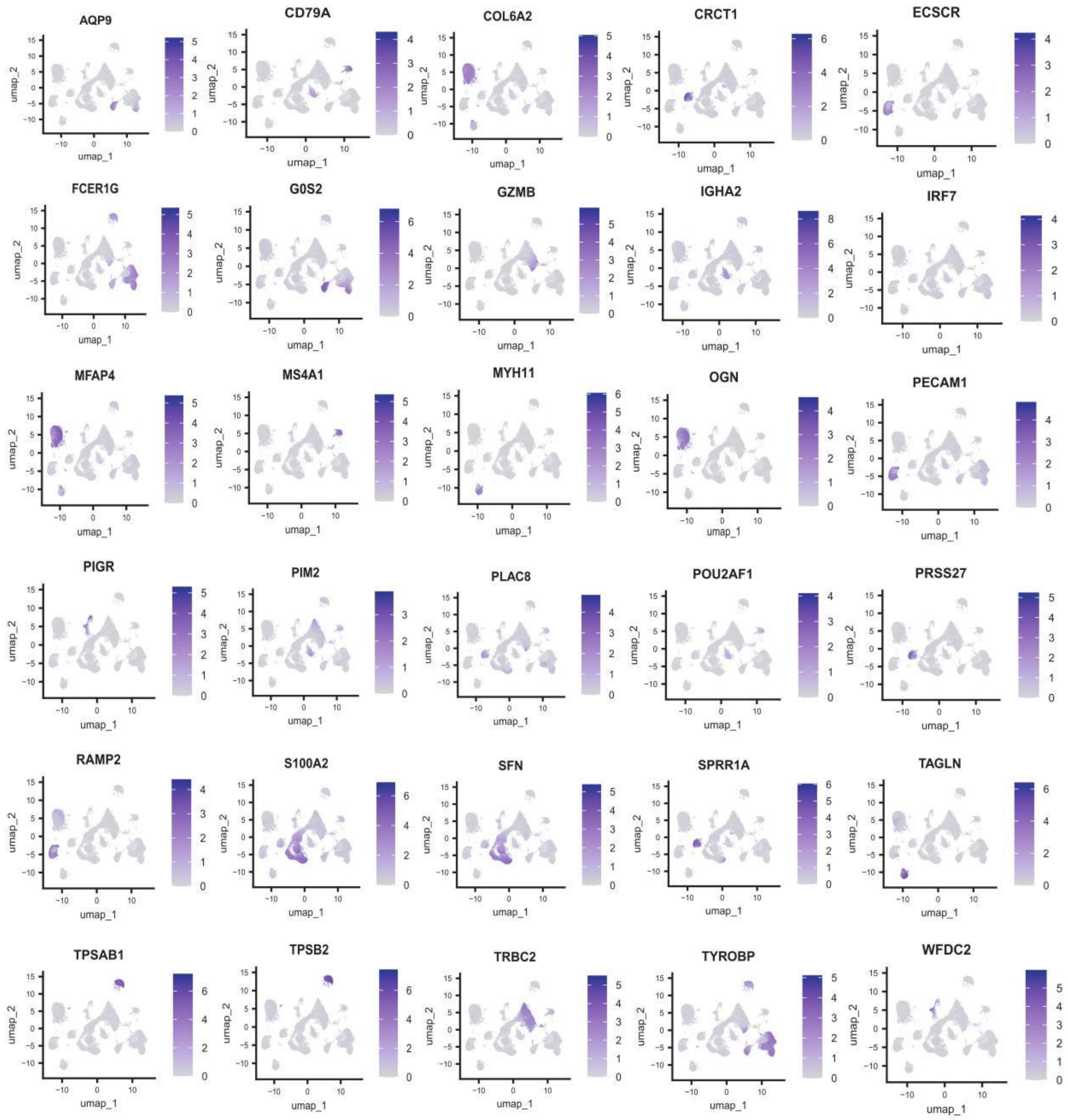

Figure S2. Expression patterns of cell-type-specific marker genes in ESCC single-cell clusters.

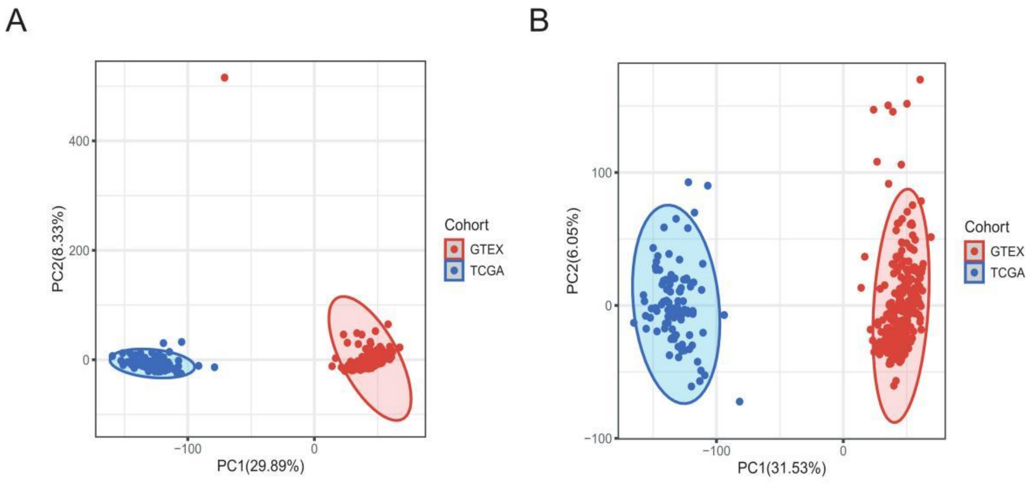

Figure S3. Principal component analysis (PCA) plots demonstrate the successful integration of transcriptomic data from the GTEx and TCGA cohorts. The distribution of samples is shown before (A) and after (B) removal of the outlier sample and correction for technical batch effects, ensuring cross-study comparability.

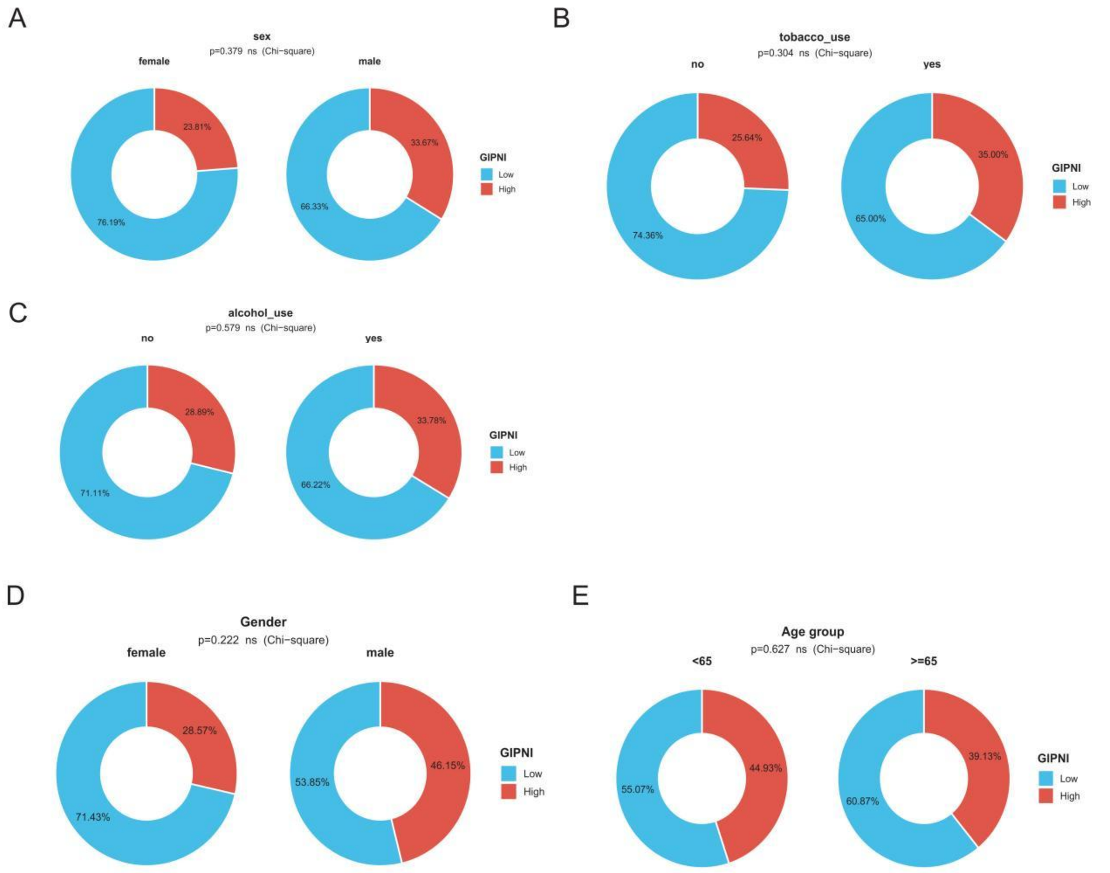

Figure S4. Donut charts illustrate the proportion of high- and low-GIPNI patients stratified by sex (A), tobacco use (B), alcohol use (C) based on GSE53624 cohort, gender, and age group based on TCGA cohort, with Chi-square test results indicating no significant bias in the index distribution across these baseline clinicopathological characteristics.

A

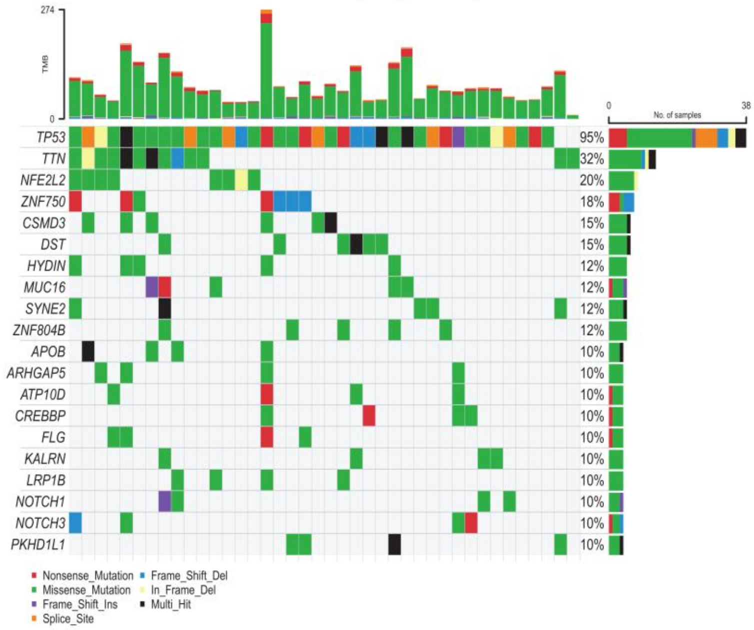

Altered in 40 (100%) of 40 samples.
274
TMB
0
0
No. of samples
38
TP53 95%
TTN 32%
NFE2L2 20%
ZNF750 18%
CSMD3 15%
DST 15%
HYDIN 12%
MUC16 12%
SYNE2 12%
ZNF804B 12%
APOB 10%
ARHGAP5 10%
ATP10D 10%
CREBBP 10%
FLG 10%
KALRN 10%
LRP1B 10%
NOTCH1 10%
NOTCH3 10%
PKHD1L1 10%
Nonsense_Mutation
Missense_Mutation
Frame_Shift_Ins
Splice_Site
Frame_Shift_Del
In_Frame_Del
Multi_Hit

B

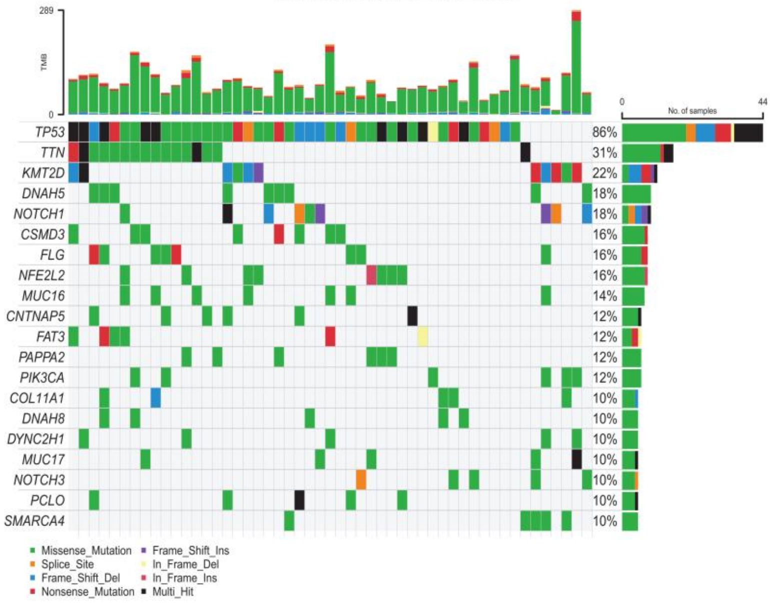

Altered in 51 (100%) of 51 samples.
289
TMB
0
0
No. of samples
44
TP53 86%
TTN 31%
KMT2D 22%
DNAH5 18%
NOTCH1 18%
CSMD3 16%
FLG 16%
NFE2L2 16%
MUC16 14%
CNTNAP5 12%
FAT3 12%
PAPPA2 12%
PIK3CA 12%
COL11A1 10%
DNAH8 10%
DYNC2H1 10%
MUC17 10%
NOTCH3 10%
PCLO 10%
SMARCA4 10%
Missense_Mutation
Splice_Site
Frame_Shift_Del
Nonsense_Mutation
Frame_Shift_Ins
In_Frame_Del
In_Frame_Ins
Multi_Hit

C

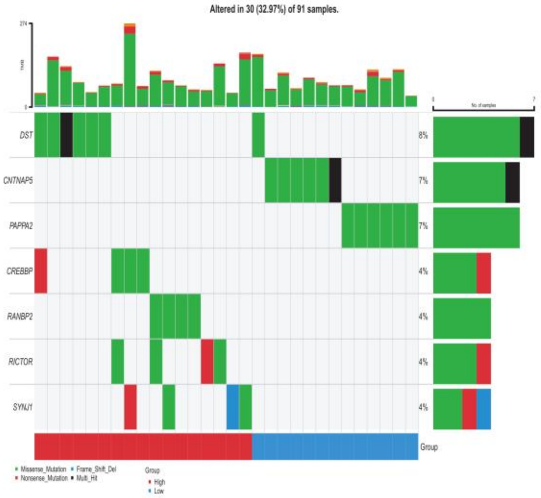

Altered in 30 (32.97%) of 91 samples.
DST 8%
CNTNAP5 7%
PAPPA2 7%
CREBBP 4%
RANBP2 4%
RICTOR 4%
SYNJ1 4%
Group
Missense_Mutation
Nonsense_Mutation
Frame_Shift_Del
Multi_Hit
Group
High
Low

Figure S5. (A) Mutational landscape of the high-GIPNI ESCC cohort. (B) Mutational landscape of the low-GIPNI ESCC cohort. (C) Differential mutation analysis between GIPNI subgroups.

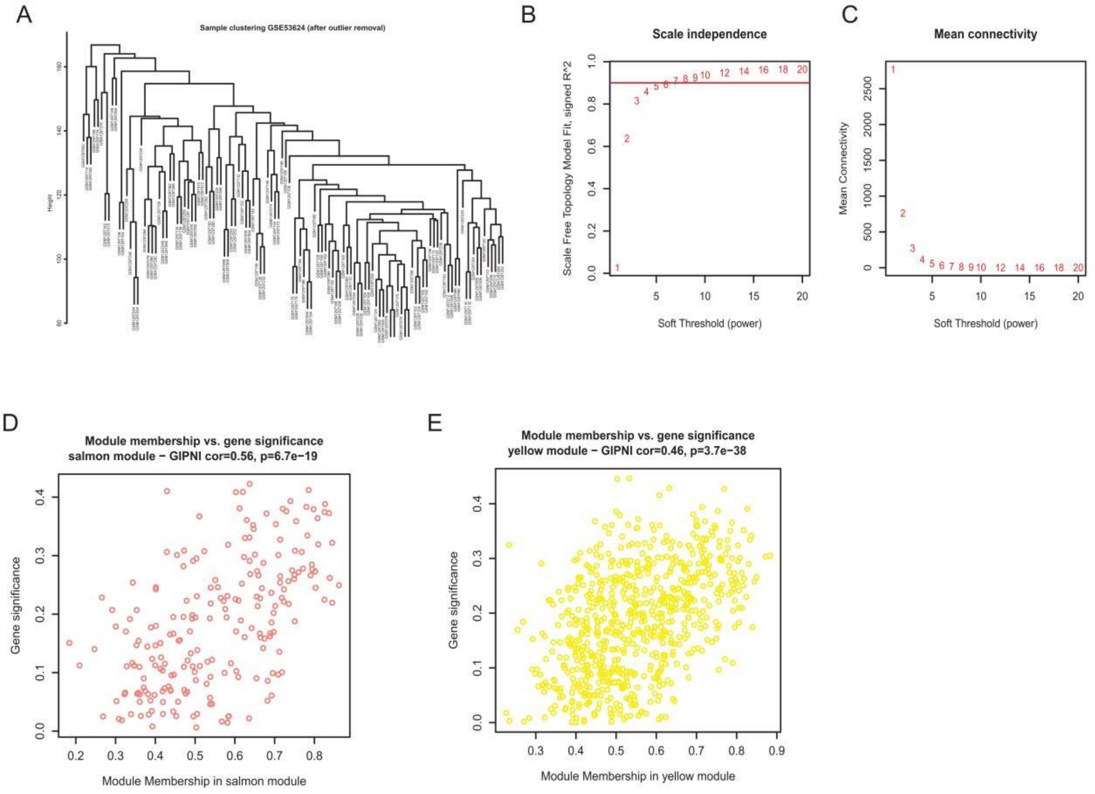

Figure S6. (A) Sample clustering and outlier detection. Dendrogram of samples from the GSE53624 dataset after outlier removal, ensuring the homogeneity of the cohort for network construction. (B, C) Determination of soft-thresholding power. Analysis of network topology showing (B) the scale-free fit index and (C) the mean connectivity for various soft-thresholding powers. (D, E) Correlation between module membership and gene significance.

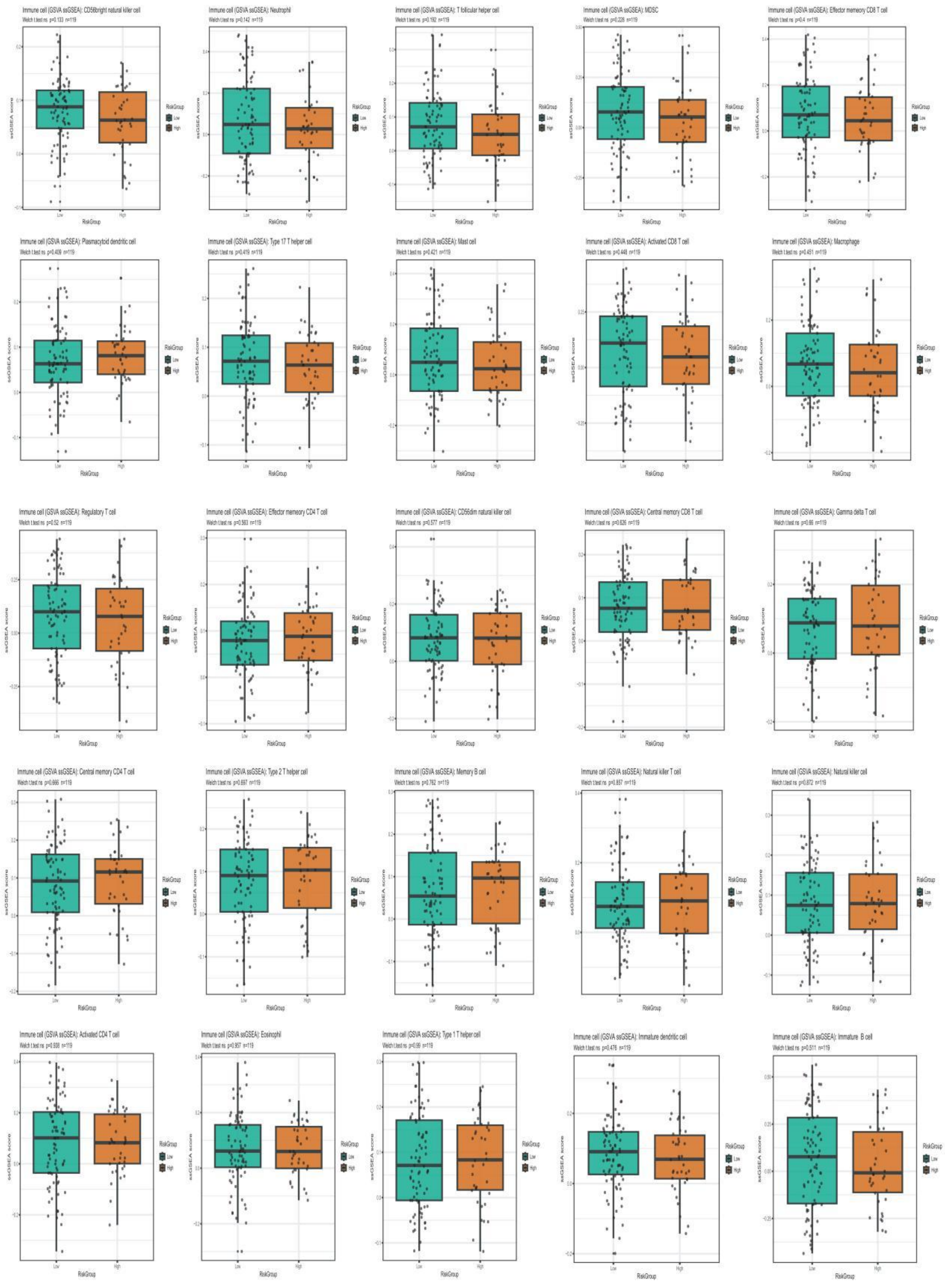

Figure S7. Landscape of immune cell infiltration between high- and low-GIPNI risk subgroups.

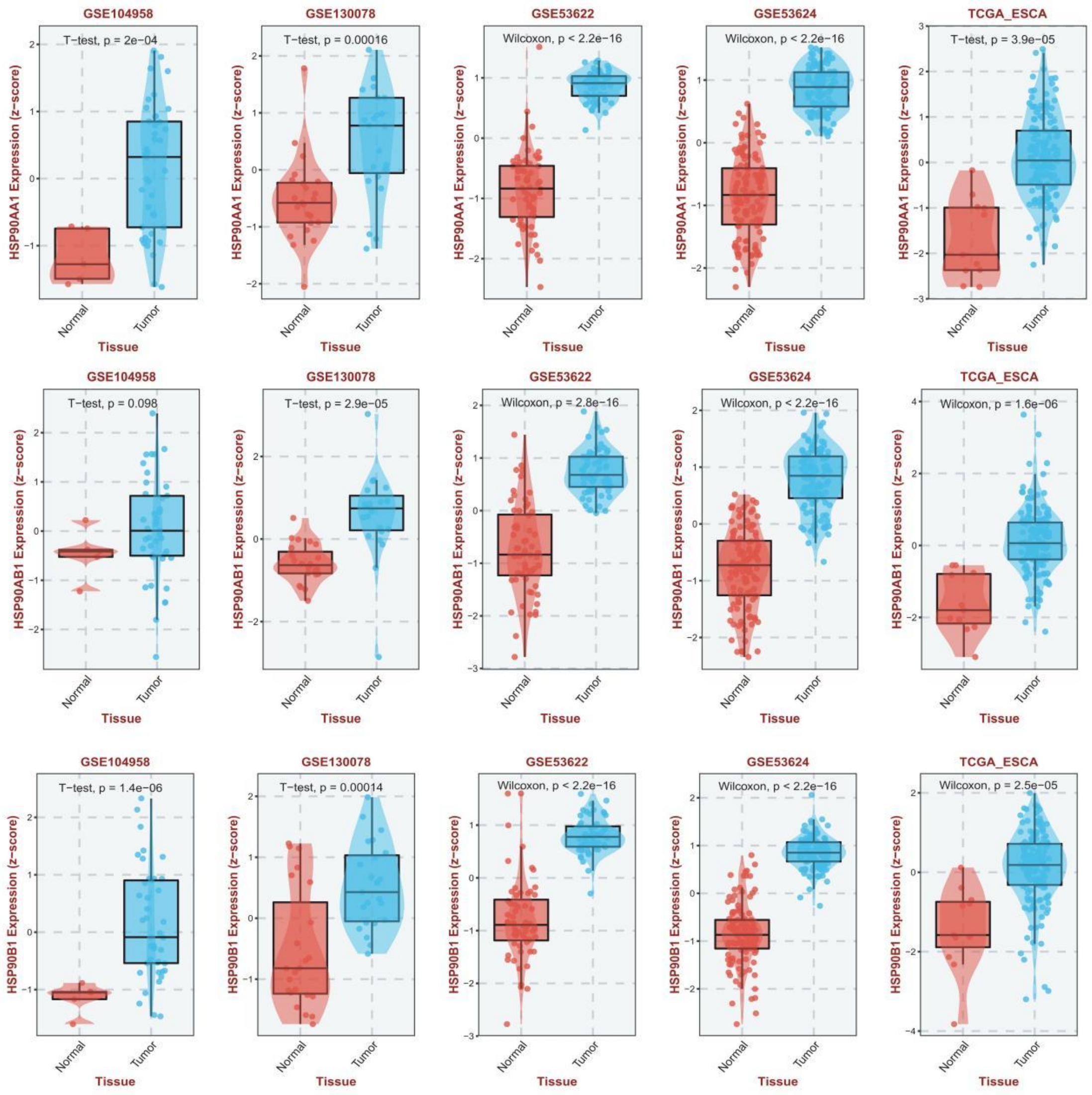

Figure S8. Violin and box plots demonstrate the consistent and significant overexpression of three representative network-perturbation genes (HSP90AA1, HSP90AB1, and HSP90B1) in tumor tissues compared with normal counterparts across five independent datasets (GSE104958, GSE130078, GSE53622, GSE53624, and TCGA-ESCA).

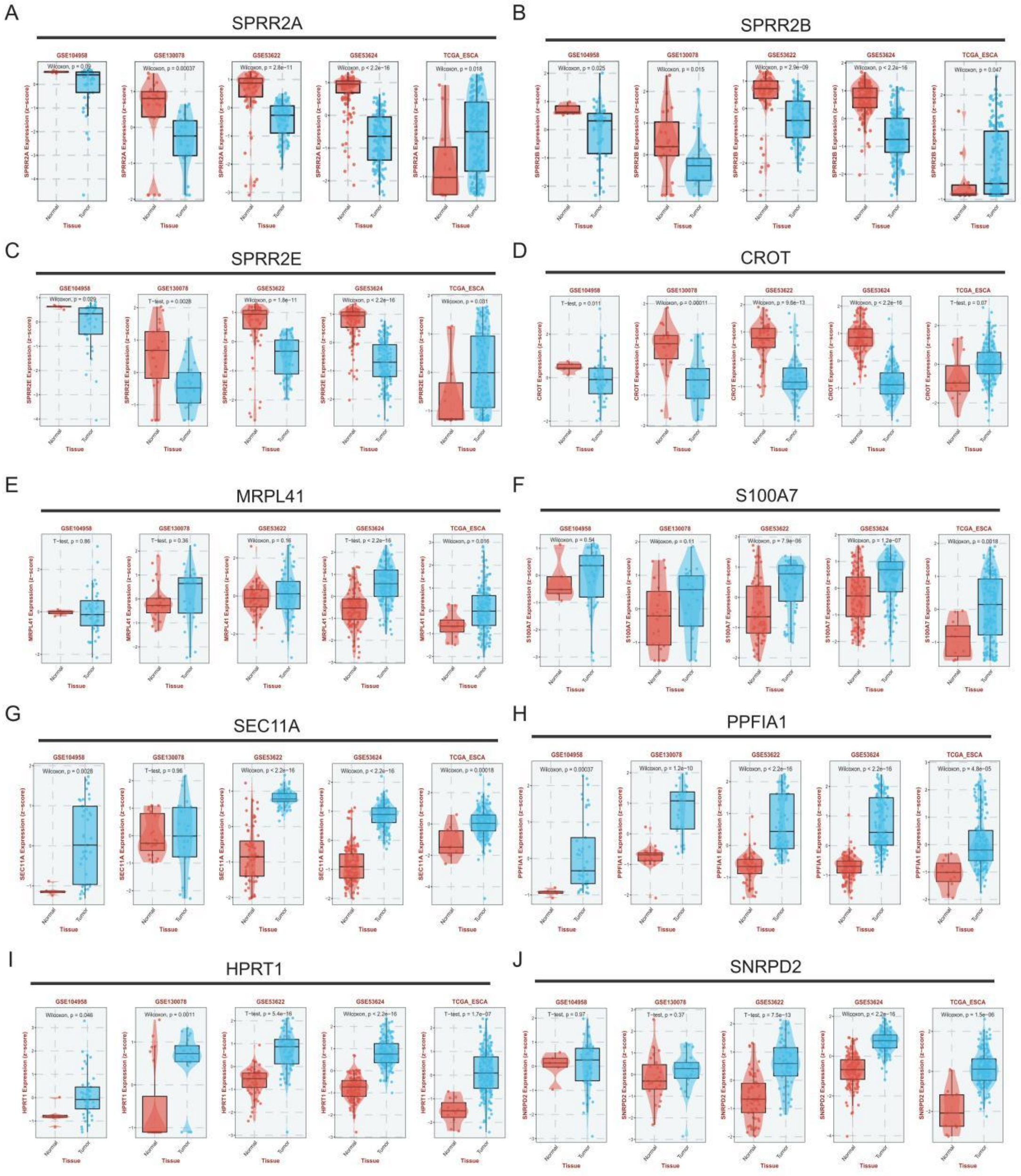

Figure S9. Violin and box plots illustrate the consistent expression dysregulation of ten key genes (SPRR2A, SPRR2B, SPRR2E, CROT, MRPL41, S100A7, SEC11A, PPFIA1, HPRT1, and SNRPD2) across five independent ESCC datasets

Supplementary tables

Table S1: SEC specific genes and dysregulated node genes.

Table S2: Gene interaction network information based on String analysis.

Table S3: Significant genes with meta-p value of GSE53624 and TCGA-ESCC cohort.
